# A preliminary inventory of the catfishes of the lower Rio Nhamundá, Brazil (Ostariophysi, Siluriformes)

**DOI:** 10.3897/BDJ.3.e4162

**Published:** 2015-04-29

**Authors:** Rupert A. Collins, Emanuell Duarte Ribeiro, Valéria Nogueira Machado, Tomas Hrbek, Izeni Pires Farias

**Affiliations:** ‡ Laboratório de Evolução e Genética Animal, Departamento de Biologia, Universidade Federal do Amazonas, Manaus, Brazil; § Programa de Pós-Graduação em Genética Conservação e Biologia Evolutiva, Instituto Nacional de Pesquisas da Amazônia, Manaus, Brazil; | Programa de Pós-Graduação em Biodiversidade e Biotecnologia, Instituto de Ciências Biológicas, Universidade Federal do Amazonas, Manaus, Brazil

**Keywords:** Biodiversity, Ichthyology, Checklist, Amazon, Guiana Shield

## Abstract

The Rio Nhamundá is a poorly-known clearwater river draining the southern Guiana Shield of Brazil. In this study we report the findings of a preliminary ichthyological survey, focusing on catfishes (Siluriformes). We identify a total of 36 species (31 genera, seven families) from the Nhamundá, including 11 species already recorded from the river. Overall, our survey results show that even rapid surveys can provide important information on Amazon fish biodiversity, suggesting potential new species, providing range extensions for nominal species, and additionally highlighting taxa in need of taxonomic revision and genetic study. As well as the traditional forms of data collected on biodiversity surveys (i.e. preserved specimen vouchers), our study also provides "new" types of data in the form of DNA barcodes and images of fishes exhibiting colouration in life, information that will be invaluable in future work addressing difficult groups.

O Rio Nhamundá é um rio de água clara, pouco conhecido, que drena parte do Escudo das Guianas em território brasileiro. Nesse estudo, nós reportamos os resultados de um levantamento ictiofaunístico preliminar dessa área, tendo como foco os bagres (Siluriformes). Nós identificamos um total de 36 espécies (31 gêneros, sete famílias) provenientes de nossa coleta, e adicionamos 11 espécies já conhecidas para o rio. De maneira geral, os resultados de nossa pesquisa mostram que mesmo levantamentos rápidos podem gerar informações importantes sobre a biodiversidade de peixes amazônicos, sugerindo potenciais espécies novas, ampliando a área de distribuição de espécies, além de apontar a necessidade de revisões taxonômicas e estudos genéticos para alguns taxa. Para além das formas tradicionais de dados coletados em pesquisas de biodiversidade (i.e. espécimes preservados), nosso estudo fornece "novas" formas de dados, como DNA barcodes e imagens com o padrão de coloração dos espécimes vivos, informações essas que serão de valor inestimável para futuros estudos que abordem grupos taxonômicos difíceis.

## Introduction

**Figure 1.**
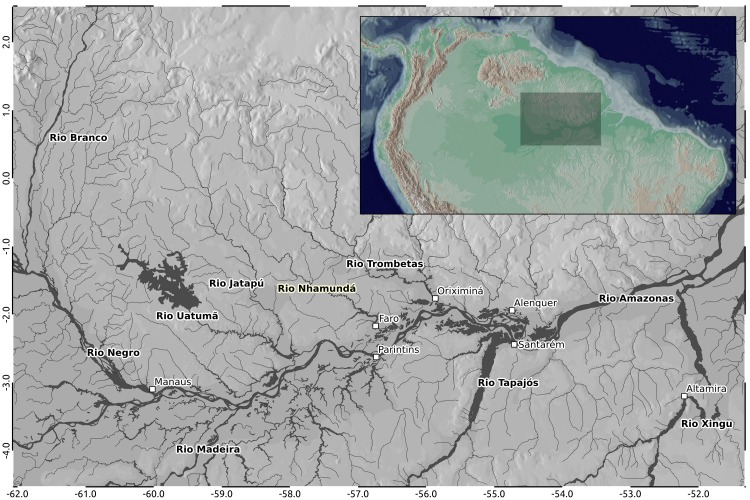
Location of the Rio Nhamundá and neighbouring rivers. Inset shows wider Amazon region. Map was created in QGIS (http://www.qgis.org/).

The Rio Nhamundá is one of the south-draining Guiana Shield tributaries of the Rio Amazonas, Brazil. The river is located between the Uatumã-Jatapú and Trombetas rivers (Fig. 1), and comprises the administrative border between Amazonas and Pará States. Chemically, the Rio Nhamundá is of the clearwater type (Carvalho de Lima and Araujo-Lima 2004), being poor in sediments, dissolved minerals, and humic compounds (Crampton 2011).

The ichthyofauna of the southern Guiana Shield rivers is poorly known in comparison to the northern Brazilian Shield rivers (Lujan and Armbruster 2011), and few scientific collections appear to have been made in the area (Vari et al. 2009). One exception is the Trombetas river, which has been extensively surveyed over several years (Ferreira 1993). Aquarium hobbyists have, however, explored parts of the Rio Nhamundá in search of new discus (Cichlidae: *Symphysodon* spp.) variants (Bleher 2006), and ornamental fishermen currently operate on the river and specialise mainly in the catching of these discus (pers. obs.).

The aim of this study is to assess our current knowledge of Siluriformes (catfishes) of the Rio Nhamundá, and to report the results of a preliminary survey of the lower reaches of the river (i.e. downstream of the last major cataracts).

## Materials and methods

### Desk survey

In order to assess the current knowledge of the catfishes of the Rio Nhamundá we carried out a survey of Web databases as well as published checklists and literature. The Web databases searched were FishBase (Froese and Pauly 2014), Catalog of Fishes (Eschmeyer 2014), and the Global Biodiversity Information Facility (GBIF; http://www.gbif.org). The FishBase and GBIF searches were conducted using the rfishbase 0.2-2 (Boettiger et al. 2012) and rgbif 0.5.0 (Chamberlain et al. 2014) packages in the R programming language (R Core Team 2014); a script to repeat these searches is presented in Suppl. material 1. The Catalog of Fishes was searched manually using their Web interface (date 05-03-14) using the terms "Nhamundá" and "Nhamunda" to avoid potential discrepancies due to the accent on the last letter. Local searches were performed on PDF copies of the Checklist of Catfishes (Ferraris 2007), the Check List of the Freshwater Fishes of South and Central America (Reis et al. 2003), and on over 3,500 locally stored articles on Neotropical ichthyology in PDF format (search script is available in Suppl. material 2). We additionally checked records for the neighbouring Trombetas and Uatumã rivers using rfishbase, rgbif, Catalog of Fishes, and Checklist of Catfishes. The database at the fish collection of the Instituto Nacional de Pesquisas da Amazônia (INPA) was also searched for catfish records from the Nhamundá drainage.

### Field sampling techniques

Fishes were collected during the November 2013 dry season using a combination of methods mostly targeting larger species: gill netting, rod and line fishing, spear fishing, hand netting, and hand catching using snorkelling equipment. Fishes were photographed alive whenever possible following the recommendations outlined in Sabaj Pérez (2009), and were then euthanised using a eugenol (clove oil) solution. Subsequent image manipulation was carried out using GIMP (http://www.gimp.org/), also following Sabaj Pérez (2009). Tissue samples—usually from muscle and fin membranes on the right-side pectoral-fin base—were taken from a subset of specimens and stored in absolute ethanol in the tissue collection of the Laboratório de Evolução e Genética Animal, Universidade Federal do Amazonas (UFAM:CTGA). Fishes to be retained as vouchers were then fixed in 10% formalin before being transferred to 70% alcohol for long-term storage in the fish collection of INPA. Other institutional abbreviations follow Sabaj Pérez (2013).

### Description of collection sites

**Figure 2.**
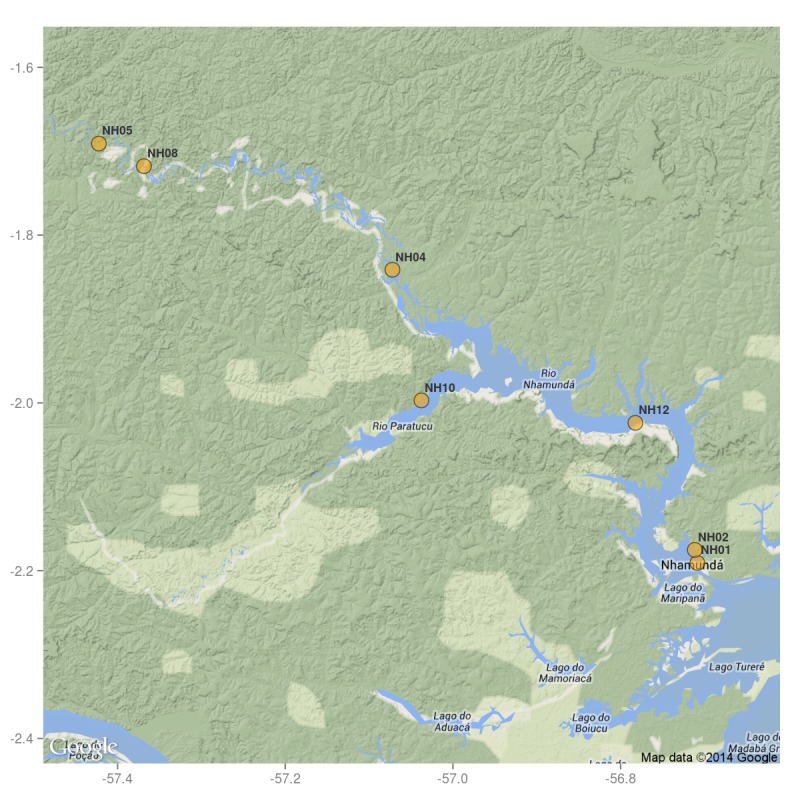
Location of sampling sites on the Rio Nhamundá. Map was created with the R package ggmap (Kahle and Wickham 2013).

Our survey of the Rio Nhamundá was undertaken at seven main sampling locations on the lower reaches of the river, and these are shown in Fig. 2. Two distinct environments were observed in our survey area: (1) a narrower, sinuous, and faster flowing upstream section encompassing sampling sites NH05, NH08, and NH04 (Fig. 3a); and (2) a wider, slow-moving, lake-like lower section of the river encompassing sampling sites NH12, NH02, and NH01 (Fig. 3b).

Sampling site NH01 comprised a small rocky promontory of a black lateritic conglomerate layer forming part of the island town of Nhamundá (Fig. 4a). The rocks were broken up into large and small boulders overlying a sand/sediment substrate (Fig. 4b), and numerous holes were present in the rocks. Water temperature was 32.0°C (daytime), and water movement was minimal. This specific habitat did not appear to be common in the immediate area, at least on the basis of the amount of rock exposed above the water. Sampling site NH02 comprised a floating wooden pen made by ornamental fishermen to house discus before resale, with terrestrial plants overhanging to provide shade for the discus. Sampling site NH12 comprised a rocky outcrop at the base of a hill (Fig. 4c). Rocks at NH12 were of a soft red lateritic type, and formed piles of large and small boulders above and below the water. Substrate at this site otherwise comprised fine sand and sediment, and water movement was minimal. Sampling site NH10 comprised a sandy beach of the Rio Paratucu, a right-hand tributary of the lower Rio Nhamundá.

Sampling site NH04 comprised a fairly typical representation of the upstream section of the river, mainly characterised by the presence of woody debris (Fig. 4d), i.e. fallen trees of varying states of decomposition (Fig. 4e). The river margin substrate was clay/soil, and water movement was moderate to strong depending on how exposed the trunks were to the main river current. Water temperature here was 28.9°C (daytime). Rocky habitats were uncommon in the upstream section of the river, with two exceptions being sampling sites NH05 (Fig. 4f) and NH08. These comprised two shallow areas of fast flowing water over a substrate of eroded bedrock and sand, forming a network of vertical fissures and crevices. Water temperature at NH05 was 28.3°C (night).

### Measurements and meristic techniques

Measurements of preserved material were taken with dial callipers to a precision of 0.1 mm. All measurements and counts for symmetrical features were taken from the left side of the specimen. For loricariids, measurements and meristic counts follow Armbruster (2003), and terminology for lateral plate rows follows Schaefer (1997). Terminology of colour pattern follows Kottelat and Freyhof (2007).

### Specimen identification

We used published literature to identify the specimens collected during our fieldwork, and we also provide here the characters found in those references that we used to make each identification. The identifications of the specimens from the INPA collection were also cross-checked. When required, we examined photographs of type material on the All Species Catfish Inventory Web page (http://acsi.acnatsci.org).

We try to keep terminology consistent across species, but also attempt to maintain a degree of agreement with original authors' work in order to aid in referring back to their characters. For example, when reporting the *Farlowella*, we refer to the "scutes" of Retzer and Page (1997) as "plates", following Schaefer (1997), but maintain reference to the "plates of second lateral row" of Retzer and Page (1997) instead of "mid-dorsal lateral plates" of Schaefer (1997).

We were conservative in applying names to the specimens collected, i.e. individuals were assigned to nominal species wherever possible, especially in cases where no modern literature for the group was available. In order to differentiate undetermined from undescribed species, we use consistent and informative tag-names following Leschen et al. (2009) for the latter, and simply "sp." for the former. If a putatively undescribed species could be determined as being allied to a nominal species, we used the "*aff.*" qualifier in order to link the record to other collection events and species. For species with significant inter-individual variation and uncertainty in the identification, we used the "*cf.*" qualifier, although we tried to avoid that wherever possible.

### Molecular methods

DNA barcodes were generated for at least one individual per species. Methodology for DNA extraction, PCR and sequencing follows Colatreli et al. (2012), but using primers FishF1 and FishR2 (Ward et al. 2005). Chromatograms were assembled in Geneious (Biomatters 2014) and aligned manually using the translated amino acid sequence. The nucleotide data was uploaded to GenBank in accordance with their DNA barcode submission tool, and comprises accession numbers KP772569:KP772605.

We also searched the Barcode of Life Data Systems (BOLD; http://www.boldsystems.org/; search term: "Genus species") and GenBank (http://www.ncbi.nlm.nih.gov/nucleotide; search term: "Genus species COI"), in order the evaluate the current coverage of deposited COI sequence data for the fishes we collected (date 20-03-14).

## Checklists

### Inventory of the catfishes of the Lower Rio Nhamundá

#### Auchenipteridae

#### Ageneiosus sp. "INPA 33873"

##### Materials

- **Type status:** Other material. **Occurrence:** catalogNumber: 33873; recordedBy: Raphael Leitão; Henrique Lazzarotto; individualCount: 1; **Taxon:** kingdom: Animalia; phylum: Chordata; class: Actinopterygii; order: Siluriformes; family: Auchenipteridae; genus: Ageneiosus; vernacularName: sp. "INPA 33873"; taxonRemarks: Undescribed species; **Location:** country: Brazil; stateProvince: Pará; locality: Lower Nhamundá River; decimalLatitude: -2.23083; decimalLongitude: -56.77306; geodeticDatum: WGS84; **Identification:** identifiedBy: R. Frederico; Rupert A. Collins; **Event:** eventDate: 2009-09-21; **Record Level:** institutionCode: INPA; basisOfRecord: PreservedSpecimen

##### Notes

This record of a single specimen from the INPA fish collection comprises an undescribed *Ageneiosus* species referred to as *A.* sp. n. "vittatus" by Akama and Ribeiro (2013).

#### Ageneiosus ucayalensis

Castelnau, 1855

##### Materials

- **Type status:** Other material. **Occurrence:** catalogNumber: 33875; recordedBy: Raphael Leitão; Henrique Lazzarotto; individualCount: 1; **Taxon:** scientificName: Ageneiosus ucayalensis Castelnau, 1855; kingdom: Animalia; phylum: Chordata; class: Actinopterygii; order: Siluriformes; family: Auchenipteridae; genus: Ageneiosus; specificEpithet: ucayalensis; scientificNameAuthorship: Castelnau, 1855; **Location:** country: Brazil; stateProvince: Pará; locality: Lower Nhamundá River; decimalLatitude: -2.23083; decimalLongitude: -56.77306; geodeticDatum: WGS84; **Identification:** identifiedBy: R. Frederico; Rupert A. Collins; **Event:** eventDate: 2009-09-21; **Record Level:** institutionCode: INPA; basisOfRecord: PreservedSpecimen

##### Notes

Record follows data from a single specimen in the collection of fishes at INPA.

#### Auchenipterichthys longimanus

(Günther, 1864)

KP772595

##### Materials

- **Type status:** Other material. **Occurrence:** catalogNumber: 43874; recordedBy: Valéria Nogueira Machado; Emanuell Duarte Ribeiro; Rupert A. Collins; individualCount: 3; otherCatalogNumbers: UFAM:CTGA:14289; UFAM:CTGA:14501; UFAM:CTGA:14502; associatedSequences: KP772595; **Taxon:** scientificName: Auchenipterichthys longimanus (Günther, 1864); kingdom: Animalia; phylum: Chordata; class: Actinopterygii; order: Siluriformes; family: Auchenipteridae; genus: Auchenipterichthys; specificEpithet: longimanus; scientificNameAuthorship: (Günther, 1864); **Location:** country: Brazil; stateProvince: Pará; locality: Lower Nhamundá River; decimalLatitude: -1.84123; decimalLongitude: -57.07212; geodeticDatum: WGS84; **Identification:** identifiedBy: Rupert A. Collins; **Event:** eventDate: 2013-11; **Record Level:** institutionCode: INPA; basisOfRecord: PreservedSpecimen

##### Notes

Identification to species level follows Ferraris et al. (2005) based on the following characters: coracoid not exposed ventrally; pelvic fin with nine branched rays; anterior teeth on premaxilla not visible when mouth closed; caudal fin emarginate; and body uniform dark colouration without dark spots.

**Figure 5.**
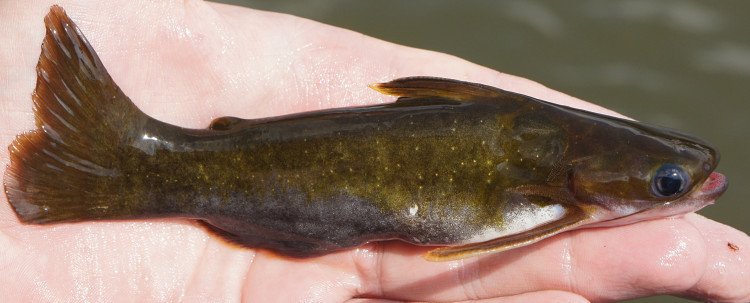
*Auchenipterichthys longimanus* live colouration (130.0 mm SL; INPA 43874; UFAM:CTGA:14501).

Three individuals were caught by hand from their lodgements in woody substrates at the margin of the main river (sampling site NH04). An example of a live specimen is pictured in Fig. 5.

#### Tatia musaica

Royero, 1992

KP772597

##### Materials

- **Type status:** Other material. **Occurrence:** catalogNumber: 43883; recordedBy: Valéria Nogueira Machado; Emanuell Duarte Ribeiro; Rupert A. Collins; individualCount: 3; otherCatalogNumbers: UFAM:CTGA:14421; UFAM:CTGA:14038; UFAM:CTGA:14039; **Taxon:** scientificName: Tatia musaica Royero, 1992; kingdom: Animalia; phylum: Chordata; class: Actinopterygii; order: Siluriformes; family: Auchenipteridae; genus: Tatia; specificEpithet: musaica; scientificNameAuthorship: Royero, 1992; **Location:** country: Brazil; stateProvince: Pará; locality: Lower Nhamundá River; decimalLatitude: -1.71782; decimalLongitude: -57.36856; geodeticDatum: WGS84; **Identification:** identifiedBy: Rupert A. Collins; identificationQualifier: aff. musaica; **Event:** eventDate: 2013-11; **Record Level:** institutionCode: INPA; basisOfRecord: PreservedSpecimen
- **Type status:** Other material. **Occurrence:** catalogNumber: 43875; recordedBy: Valéria Nogueira Machado; Emanuell Duarte Ribeiro; Rupert A. Collins; individualCount: 4; otherCatalogNumbers: UFAM:CTGA:14507; UFAM:CTGA:14508; UFAM:CTGA:14040; UFAM:CTGA:14041; associatedSequences: KP772597; **Taxon:** scientificName: Tatia musaica Royero, 1992; kingdom: Animalia; phylum: Chordata; class: Actinopterygii; order: Siluriformes; family: Auchenipteridae; genus: Tatia; specificEpithet: musaica; scientificNameAuthorship: Royero, 1992; **Location:** country: Brazil; stateProvince: Pará; locality: Lower Nhamundá River; decimalLatitude: -1.84123; decimalLongitude: -57.07212; geodeticDatum: WGS84; **Identification:** identifiedBy: Rupert A. Collins; identificationQualifier: aff. musaica; **Event:** eventDate: 2013-11; **Record Level:** institutionCode: INPA; basisOfRecord: PreservedSpecimen
- **Type status:** Other material. **Occurrence:** catalogNumber: 43867; recordedBy: Valéria Nogueira Machado; Emanuell Duarte Ribeiro; Rupert A. Collins; individualCount: 9; **Taxon:** scientificName: Tatia musaica Royero, 1992; kingdom: Animalia; phylum: Chordata; class: Actinopterygii; order: Siluriformes; family: Auchenipteridae; genus: Tatia; specificEpithet: musaica; scientificNameAuthorship: Royero, 1992; **Location:** country: Brazil; stateProvince: Pará; locality: Lower Nhamundá River; decimalLatitude: -2.02386; decimalLongitude: -56.78235; geodeticDatum: WGS84; **Identification:** identifiedBy: Rupert A. Collins; identificationQualifier: aff. musaica; **Event:** eventDate: 2013-11; **Record Level:** institutionCode: INPA; basisOfRecord: PreservedSpecimen
- **Type status:** Other material. **Occurrence:** catalogNumber: 14031; 14032; 14033; recordedBy: Tomas Hrbek; José Gregorio Martínez; Joiciane Gonçalves Farias; individualCount: 3; associatedSequences: KP772569; KP772570; KP772571; **Taxon:** scientificName: Tatia musaica Royero, 1992; kingdom: Animalia; phylum: Chordata; class: Actinopterygii; order: Siluriformes; family: Auchenipteridae; genus: Tatia; specificEpithet: musaica; scientificNameAuthorship: Royero, 1992; **Location:** country: Colombia; stateProvince: Guainia; locality: Lower Atabapo River; decimalLatitude: 4.02883; decimalLongitude: -67.70458; geodeticDatum: WGS84; **Identification:** identifiedBy: Rupert A. Collins; **Event:** eventDate: 2014-05; **Record Level:** institutionCode: UFAM; collectionCode: CTGA; basisOfRecord: PreservedSpecimen
- **Type status:** Other material. **Occurrence:** catalogNumber: 35086; recordedBy: Raphael Leitão; Henrique Lazzarotto; individualCount: 36; **Taxon:** scientificName: Tatia musaica Royero, 1992; kingdom: Animalia; phylum: Chordata; class: Actinopterygii; order: Siluriformes; family: Auchenipteridae; genus: Tatia; specificEpithet: musaica; scientificNameAuthorship: Royero, 1992; **Location:** country: Brazil; stateProvince: Pará; locality: Lower Nhamundá River; decimalLatitude: -2.00111; decimalLongitude: -56.51889; geodeticDatum: WGS84; **Identification:** identifiedBy: Raphael Leitão; Rupert A. Collins; identificationQualifier: aff. musaica; **Event:** eventDate: 2009-09-22; **Record Level:** institutionCode: INPA; basisOfRecord: PreservedSpecimen

##### Notes

Identification as a possibly undescribed species similar to *Tatia musaica*, follows Royero (1992), Sarmento-Soares and Martins-Pinheiro (2008), Vari and Ferraris (2013), and Vari and Calegari (2014) based on the following characters: strongly contrasting dorsal (black) and ventral (white) colour pattern, with irregular lateral pigmentation extending nearly to ventrum; pigmentation extending onto caudal fin lobes as medial stripes; pigmented dorsal-fin spine; bifid third nuchal plate; and long post-cleithral process surpassing the nuchal plates.

Specifically, our specimens differ from *T. musaica* as described by Royero (1992) (images can be found on the All Species Catfish Inventory Web page; http://acsi.acnatsci.org/base/image_list.html?mode=genus&genus=Tatia) in at least two aspects: unpigmented area dorso-posteriorly to the orbit (slightly elongated and smaller than orbit diameter); and third nuchal plate larger and unpigmented (including some of the surrounding skin). Our examination of topotypic *T. musaica* material from the Río Atabapo of Colombia/Venezuela confirm these differences, in addition to a 5.2% COI sequence divergence (32 mutations over 615 bp). Our Nhamundá specimens are also similar to *Tatia melanoleuca* as described by Vari and Calegari (2014), but differ from this species mainly in respect to the distribution of dark pigmentation on the caudal fin, dorsal-fin spine, and lateral surfaces.

**Figure 6.**
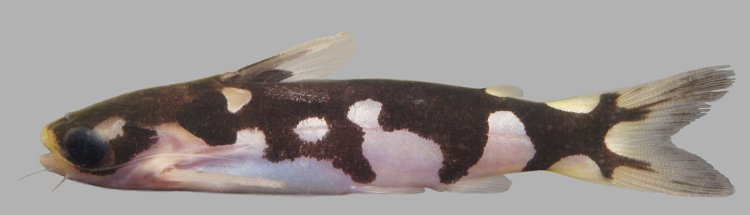
Tatia aff. musaica live colouration (47.8 mm SL; INPA 43875; UFAM:CTGA:14508).

Sixteen individuals were caught either by hand from their lodgements in woody substrates (sampling sites NH04 and NH08), or more effectively using a hand net at the surface after attracting insects—on which they were feeding—with a light (sampling site NH12); under such conditions they appeared abundant on the lower Nhamundá. An example of a live specimen is pictured in Fig. 6.

Two lots of *Centromochlus* from the INPA fish collection were collected from the Rio Nhamundá. Both were out on loan at the time of this study and could not be examined here, but the first—36 specimens of *Centromochlus* sp. "orca" (INPA 35086)—contains a fish clearly conspecific with our T. aff. musaica based on the assigned tag name, and so we have included this lot under our T. aff. musaica. We were unsure of the identity of the second—a record of a *Centromochlus* sp. "pigmento" (INPA 35087) referred to by Sarmento-Soares et al. (2013) as *Centromochlus* sp.—and so have not included this species in the checklist.

#### Tatia nigra

Sarmento-Soares & Martins-Pinheiro, 2008

KP772596

##### Materials

- **Type status:** Other material. **Occurrence:** catalogNumber: 43876; recordedBy: Valéria Nogueira Machado; Emanuell Duarte Ribeiro; Rupert A. Collins; individualCount: 4; otherCatalogNumbers: UFAM:CTGA:14503; UFAM:CTGA:14504; UFAM:CTGA:14505; UFAM:CTGA:14506; associatedSequences: KP772596; **Taxon:** scientificName: Tatia nigra Sarmento-Soares & Martins-Pinheiro, 2008; kingdom: Animalia; phylum: Chordata; class: Actinopterygii; order: Siluriformes; family: Auchenipteridae; genus: Tatia; specificEpithet: nigra; scientificNameAuthorship: Sarmento-Soares & Martins-Pinheiro, 2008; **Location:** country: Brazil; stateProvince: Pará; locality: Lower Nhamundá River; decimalLatitude: -1.84123; decimalLongitude: -57.07212; geodeticDatum: WGS84; **Identification:** identifiedBy: Rupert A. Collins; **Event:** eventDate: 2013-11; **Record Level:** institutionCode: INPA; basisOfRecord: PreservedSpecimen

##### Notes

Identification to species level follows Sarmento-Soares and Martins-Pinheiro (2008) based on the following characters: short post-cleithral process (about 60% of head length) not reaching vertical through origin of dorsal fin; and body colouration dark brown.

**Figure 7.**
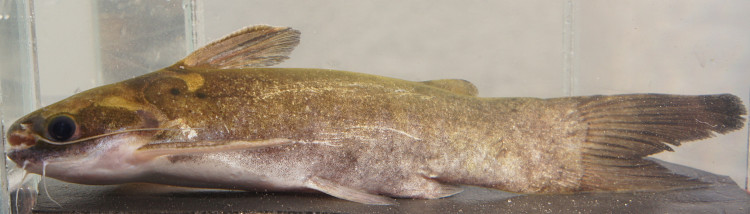
*Tatia nigra* live colouration (101.0 mm SL; INPA 43876; UFAM:CTGA:14503).

Four individuals were caught by hand from their lodgements in woody substrates at the margin of the main river (sampling site NH04). The species was also observed in rocky habitats (sampling sites NH08 and NH12), but were more difficult to catch in this situation. An example of a live specimen is pictured in Fig. 7.

#### Trachycorystes trachycorystes

(Valenciennes, 1840)

KP772586

##### Materials

- **Type status:** Other material. **Occurrence:** catalogNumber: 43897; recordedBy: Valéria Nogueira Machado; Emanuell Duarte Ribeiro; Rupert A. Collins; individualCount: 2; otherCatalogNumbers: UFAM:CTGA:14428; UFAM:CTGA:14429; associatedSequences: KP772586; **Taxon:** scientificName: Trachycorystes trachycorystes (Valenciennes, 1840); kingdom: Animalia; phylum: Chordata; class: Actinopterygii; order: Siluriformes; family: Auchenipteridae; genus: Trachycorystes; specificEpithet: trachycorystes; scientificNameAuthorship: (Valenciennes, 1840); **Location:** country: Brazil; stateProvince: Pará; locality: Lower Nhamundá River; decimalLatitude: -1.67511; decimalLongitude: -57.47678; geodeticDatum: WGS84; **Identification:** identifiedBy: Rupert A. Collins; **Event:** eventDate: 2013-11; **Record Level:** institutionCode: INPA; basisOfRecord: PreservedSpecimen

##### Notes

Identification to species level follows Britski and Akama (2011) based on the following characters: lower jaw prognathus; skull roof covered by thin integument; inner mental barbel reaching base of outer mental barbel; dorsal-fin spine serrated only along anterior margin; and caudal fin emarginate (our specimens had suffered damage to the lobes of the caudal fin, presumably due to piranhas).

**Figure 8.**
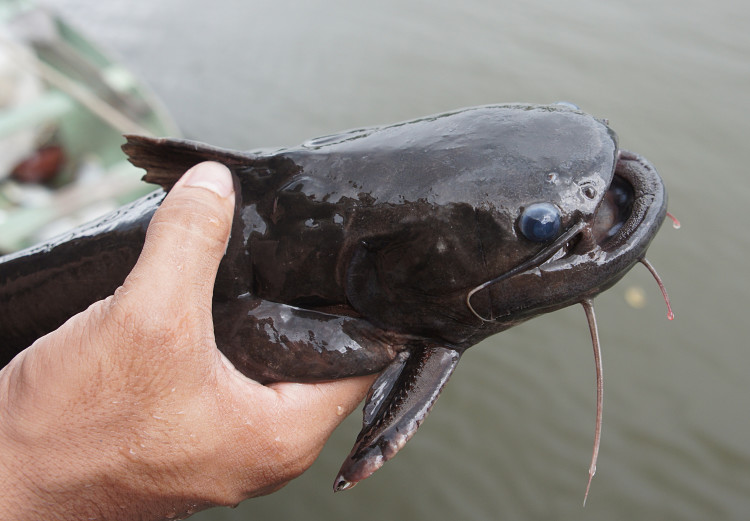
*Trachycorystes trachycorystes* live colouration (305.0 mm SL; INPA 43897; UFAM:CTGA:14429). Image flipped horizontally.

Two individuals were caught at night using gill nets set in the margins of the main river. An example of a live specimen is pictured in Fig. 8.

#### Doradidae

#### Astrodoras asterifrons

(Kner, 1853)

KP772601

##### Materials

- **Type status:** Other material. **Occurrence:** catalogNumber: 43868; recordedBy: Valéria Nogueira Machado; Emanuell Duarte Ribeiro; Rupert A. Collins; individualCount: 2; otherCatalogNumbers: UFAM:CTGA:14539; UFAM:CTGA:14540; associatedSequences: KP772601; **Taxon:** scientificName: Astrodoras asterifrons (Kner, 1853); kingdom: Animalia; phylum: Chordata; class: Actinopterygii; order: Siluriformes; family: Doradidae; genus: Astrodoras; specificEpithet: asterifrons; scientificNameAuthorship: (Kner, 1853); **Location:** country: Brazil; stateProvince: Pará; locality: Lower Nhamundá River; decimalLatitude: -2.02386; decimalLongitude: -56.78235; geodeticDatum: WGS84; **Identification:** identifiedBy: Rupert A. Collins; **Event:** eventDate: 2013-11; **Record Level:** institutionCode: INPA; basisOfRecord: PreservedSpecimen

##### Notes

Identification to species level follows Sousa (2010) based on the following characters: flattened body shape; upper limit of pre-opercular canal not reaching lateral border of the cranium; seven branched rays in the dorsal lobe of the caudal fin; dorsal margin of the orbit high; procurrent rays of the caudal fin expanded into bony shields; and diverticula of the swim bladder simple, with tapered rear end.

**Figure 9.**
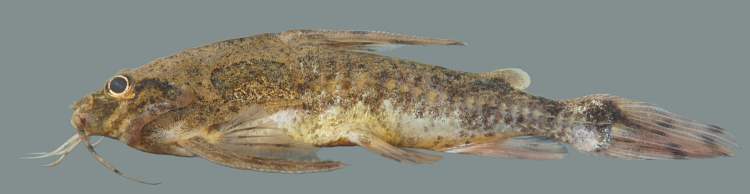
*Astrodoras asterifrons* live colouration (69.7 mm SL; INPA 43868; UFAM:CTGA:14540)

Two individuals were caught by hand-net at night over a sandy/silty substrate (sampling site NH12). An example of a live specimen is pictured in Fig. 9.

#### Hassar orestis

(Steindachner, 1875)

##### Notes

Record follows data from Birindelli et al. (2011).

#### Ossancora asterophysa

Birindelli & Sabaj Pérez, 2011

##### Notes

Record follows data from Birindelli and Sabaj Pérez (2011).

#### Scorpiodoras heckelii

(Kner, 1855)

KP772600

##### Materials

- **Type status:** Other material. **Occurrence:** catalogNumber: 43872; recordedBy: Valéria Nogueira Machado; Emanuell Duarte Ribeiro; Rupert A. Collins; individualCount: 1; otherCatalogNumbers: UFAM:CTGA:14538; associatedSequences: KP772600; **Taxon:** scientificName: Scorpiodoras heckelii (Kner, 1855); kingdom: Animalia; phylum: Chordata; class: Actinopterygii; order: Siluriformes; family: Doradidae; genus: Scorpiodoras; specificEpithet: heckelii; scientificNameAuthorship: (Kner, 1855); **Location:** country: Brazil; stateProvince: Pará; locality: Lower Nhamundá River; decimalLatitude: -1.99702; decimalLongitude: -57.03758; geodeticDatum: WGS84; **Identification:** identifiedBy: Rupert A. Collins; **Event:** eventDate: 2013-11; **Record Level:** institutionCode: INPA; basisOfRecord: PreservedSpecimen

##### Notes

Identification to species level for this specimen could not be adequately made using morphological characters due to the small size of the immature specimen. However, rather than exclude the individual, we compared the DNA barcodes to the COI sequences presented by Arce et al. (2013) in their doradid phylogeny. The specimen clustered with, and was 1.36% divergent from (seven mutations in 516 bp), *Scorpiodoras heckelii* (GenBank KC555695), and is most likely conspecific given the known distribution of the species and its congeners (Sousa and Birindelli 2011).

**Figure 10.**
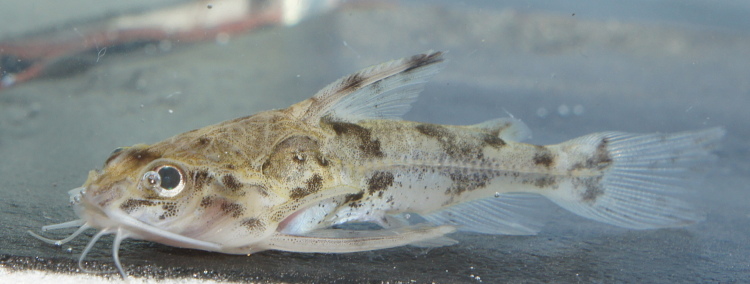
*Scorpiodoras heckelii* juvenile, live colouration (30.1 mm SL; INPA 43872; UFAM:CTGA:14538).

One individual was caught by hand-net on the Rio Paratucu (sampling site NH10). This specimen is pictured in Fig. 10.

#### Heptapteridae

#### Goeldiella eques

(Müller & Troschel, 1849)

KP772599

##### Materials

- **Type status:** Other material. **Occurrence:** catalogNumber: 43873; recordedBy: Valéria Nogueira Machado; Emanuell Duarte Ribeiro; Rupert A. Collins; individualCount: 1; otherCatalogNumbers: UFAM:CTGA:14537; associatedSequences: KP772599; **Taxon:** scientificName: Goeldiella eques (Müller & Troschel, 1849); kingdom: Animalia; phylum: Chordata; class: Actinopterygii; order: Siluriformes; family: Heptapteridae; genus: Goeldiella; specificEpithet: eques; scientificNameAuthorship: (Müller & Troschel, 1849); **Location:** country: Brazil; stateProvince: Pará; locality: Lower Nhamundá River; decimalLatitude: -1.99702; decimalLongitude: -57.03758; geodeticDatum: WGS84; **Identification:** identifiedBy: Rupert A. Collins; **Event:** eventDate: 2013-11; **Record Level:** institutionCode: INPA; basisOfRecord: PreservedSpecimen

##### Notes

Identification to species level follows Eigenmann and Norris (1900) and Eigenmann (1912) based on the following characters: rounded caudal fin with larger lower lobe; distinct cranial fontanelle; maxillary barbels long, extending to caudal (extended only to caudal peduncle in our specimen); dorsal spine notched anteriorly; dark stripe along lateral line (in our specimen this comprised just a elongated blotch under the dorsal fin); base of caudal with dark bar; and obliquely angled dark saddle behind head (from dorsal insertion to base of opercle); and body and fins irregularly mottled.

**Figure 11.**
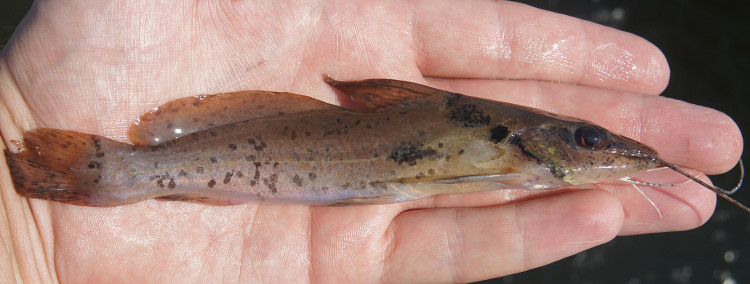
*Goeldiella eques* live colouration (134.0 mm SL; INPA 43873; UFAM:CTGA:14537).

One individual was caught by hand-net on the Rio Paratucu (sampling site NH10), and delivered a painful sting, confirming that many heptapterids are venomous (Wright 2009). This specimen is pictured in Fig. 11.

#### Pimelodella sp.

KP772572

##### Materials

- **Type status:** Other material. **Occurrence:** catalogNumber: 43890; recordedBy: Valéria Nogueira Machado; Emanuell Duarte Ribeiro; Rupert A. Collins; individualCount: 1; otherCatalogNumbers: UFAM:CTGA:14290; associatedSequences: KP772572; **Taxon:** scientificName: Pimelodella; kingdom: Animalia; phylum: Chordata; class: Actinopterygii; order: Siluriformes; family: Heptapteridae; genus: Pimelodella; taxonRemarks: Species undetermined; **Location:** country: Brazil; stateProvince: Pará; locality: Lower Nhamundá River; decimalLatitude: -1.6909; decimalLongitude: -57.42231; geodeticDatum: WGS84; **Identification:** identifiedBy: Rupert A. Collins; **Event:** eventDate: 2013-11; **Record Level:** institutionCode: INPA; basisOfRecord: PreservedSpecimen

##### Notes

Tentative identification to genus level follows Eigenmann and Eigenmann (1890) and Eigenmann (1917) based on the following characters: occipital process narrow, reaching dorsal plate; fontanel continued to base of occipital process, with bridge above the posterior margin of the eye; dorsal-fin and pectoral-fin spines strong; humeral process spine-like; and dorsal fin with six branched rays.

Given the large diversity of the group, and the paucity of modern treatments dealing with heptapterids, we are currently unable to identify this fish to species level, and our genus identification is tentative. Important characters include the caudal fin lobes of approximately equal length, maxillary barbels not surpassing anal fin (left barbel is damaged in our specimen), the complete lack of dark longitudinal stripe, the wedge-shaped mark on the dorsal-fin, and the dark saddle anterior to the dorsal fin.

**Figure 12.**
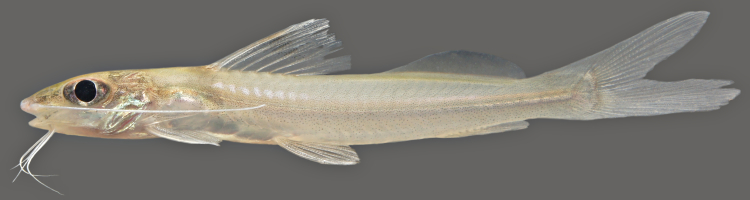
*Pimelodella* sp. live colouration (48.9 mm SL; INPA 43890; UFAM:CTGA:14290).

One individual was caught by hand-net on a sandy beach habitat (adjacent to sampling site NH05). This specimen is pictured in Fig. 12.

#### Loricariidae

#### Hypoptopomatinae

#### Hypoptopoma incognitum

Aquino & Schaefer, 2010

KP772573

##### Materials

- **Type status:** Other material. **Occurrence:** catalogNumber: 43865; recordedBy: Valéria Nogueira Machado; Emanuell Duarte Ribeiro; Rupert A. Collins; individualCount: 13; otherCatalogNumbers: UFAM:CTGA:14306; UFAM:CTGA:14307; UFAM:CTGA:14308; UFAM:CTGA:14309; UFAM:CTGA:14310; associatedSequences: KP772573; **Taxon:** scientificName: Hypoptopoma incognitum Aquino & Schaefer, 2010; kingdom: Animalia; phylum: Chordata; class: Actinopterygii; order: Siluriformes; family: Loricariidae; genus: Hypoptopoma; specificEpithet: incognitum; scientificNameAuthorship: Aquino & Schaefer, 2010; **Location:** country: Brazil; stateProvince: Pará; locality: Lower Nhamundá River; decimalLatitude: -2.17525; decimalLongitude: -56.7115; geodeticDatum: WGS84; **Identification:** identifiedBy: Rupert A. Collins; **Event:** eventDate: 2013-11; **Record Level:** institutionCode: INPA; basisOfRecord: PreservedSpecimen

##### Notes

Identification to species level follows Aquino and Schaefer (2010) based on the following characters: flattened head with eyes placed ventrolaterally and visible from below; laterally expanded nuchal plate; six pairs of lateral abdominal plates posterior to coracoids; thoracic plates present; three midventral plates between cleithral posterior process and first plate of ventral series; anal shield composed of single plate; second infraorbital laterally contacting to two ventral dermal plates; patch of odontodes present on anterolateral aspect of cleithrum at opening to branchial chamber; and caudal fin with series of around three dark bands (irregular in our specimens).

**Figure 13.**
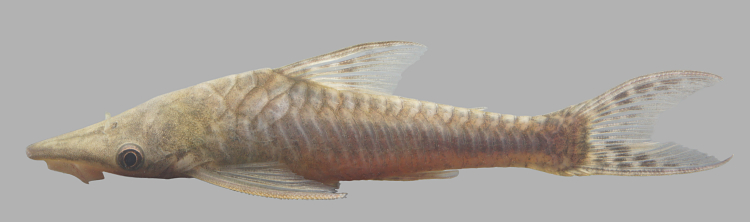
*Hypoptopoma incognitum* live colouration (83.9 mm SL; INPA 43865; UFAM:CTGA:14310).

Thirteen individuals were caught by hand from submerged terrestrial vegetation (sampling site NH02). An example of a live specimen is pictured in Fig. 13.

#### Hypostominae

#### Ancistrus dolichopterus

Kner, 1854

KP772578

KP772593

##### Materials

- **Type status:** Other material. **Occurrence:** catalogNumber: 43877; recordedBy: Valéria Nogueira Machado; Emanuell Duarte Ribeiro; Rupert A. Collins; individualCount: 10; otherCatalogNumbers: UFAM:CTGA:14320; UFAM:CTGA:14321; UFAM:CTGA:14322; UFAM:CTGA:14323; UFAM:CTGA:14324; UFAM:CTGA:14489; UFAM:CTGA:14490; UFAM:CTGA:14491; UFAM:CTGA:14492; UFAM:CTGA:14493; associatedSequences: KP772578; KP772593; **Taxon:** scientificName: Ancistrus dolichopterus Kner, 1854; kingdom: Animalia; phylum: Chordata; class: Actinopterygii; order: Siluriformes; family: Loricariidae; genus: Ancistrus; specificEpithet: dolichopterus; scientificNameAuthorship: Kner, 1854; **Location:** country: Brazil; stateProvince: Pará; locality: Lower Nhamundá River; decimalLatitude: -1.84123; decimalLongitude: -57.07212; geodeticDatum: WGS84; **Identification:** identifiedBy: Rupert A. Collins; **Event:** eventDate: 2013-11; **Record Level:** institutionCode: INPA; basisOfRecord: PreservedSpecimen
- **Type status:** Other material. **Occurrence:** catalogNumber: 43861; recordedBy: Valéria Nogueira Machado; Emanuell Duarte Ribeiro; Rupert A. Collins; individualCount: 1; otherCatalogNumbers: UFAM:CTGA:14549; **Taxon:** scientificName: Ancistrus dolichopterus Kner, 1854; kingdom: Animalia; phylum: Chordata; class: Actinopterygii; order: Siluriformes; family: Loricariidae; genus: Ancistrus; specificEpithet: dolichopterus; scientificNameAuthorship: Kner, 1854; **Location:** country: Brazil; stateProvince: Pará; locality: Lower Nhamundá River; decimalLatitude: -2.19081; decimalLongitude: -56.7084; geodeticDatum: WGS84; **Identification:** identifiedBy: Rupert A. Collins; **Event:** eventDate: 2013-11; **Record Level:** institutionCode: INPA; basisOfRecord: PreservedSpecimen

##### Notes

Identification to species level follows Armbruster (2004), and Kner (1854) based on the following characters: three rows of lateral plates on the caudal peduncle; snout naked with fleshy tentacles lacking odontodes; 8-9 branched dorsal-fin rays (three individuals with nine rays and eight individuals with eight rays); and black colour with small white dots (apparent on body and fins in life, but only apparent on abdomen in preserved material).

We note that *Ancistrus*, and particularly the Amazonian species, are a group in dire need of taxonomic revision.

**Figure 14.**
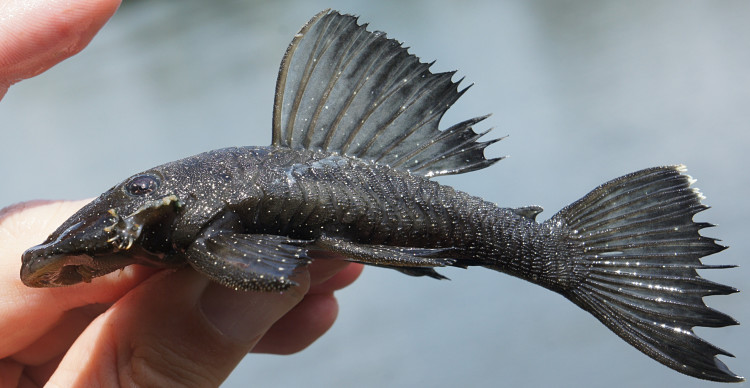
*Ancistrus dolichopterus* live colouration (87.8 mm SL; INPA 43877; UFAM:CTGA:14490).

Eleven individuals were caught by hand on both woody (sampling site NH04) and rocky substrates (sampling site NH01). The species appeared abundant throughout the river. An example of a live specimen is pictured in Fig. 14.

#### Ancistrus sp. "INPA 43862"

KP772604

##### Materials

- **Type status:** Other material. **Occurrence:** catalogNumber: 43862; recordedBy: Valéria Nogueira Machado; Emanuell Duarte Ribeiro; Rupert A. Collins; individualCount: 2; otherCatalogNumbers: UFAM:CTGA:14547; UFAM:CTGA:14548; associatedSequences: KP772604; **Taxon:** scientificName: Ancistrus; kingdom: Animalia; phylum: Chordata; class: Actinopterygii; order: Siluriformes; family: Loricariidae; genus: Ancistrus; taxonRank: genus; vernacularName: sp. "INPA 43862"; taxonRemarks: Possible undescribed species; **Location:** country: Brazil; stateProvince: Pará; locality: Lower Nhamundá River; decimalLatitude: -2.19081; decimalLongitude: -56.7084; geodeticDatum: WGS84; **Identification:** identifiedBy: Rupert A. Collins; **Event:** eventDate: 2013-11; **Record Level:** institutionCode: INPA; basisOfRecord: PreservedSpecimen

##### Notes

Following Armbruster (2004), Kner (1854), Muller et al. (1994), Günther (1864), Pellegrin (1912), and Eigenmann (1912) we report the following combination of characters allowing a genus-level identification only: three rows of lateral plates on the caudal peduncle; snout naked with fleshy tentacles lacking odontodes; body and head wide and extremely flattened; snout long and pointed; seven branched dorsal-fin rays; six branched pectoral-fin rays; eyes large (orbit diameter approximately 20% of HL) and situated high on the head; narrow gill openings; and colouration black, with small yellow-white dots in life.

Among the superficially similar nominal *Ancistrus*—e.g. *A. dolichopterus* Kner, 1854, *A. hoplogenys* (Günther, 1864), *A. leucostictus* (Günther, 1864), *A. lithurgicus* Eigenmann, 1912, *A. macrophthalmus* (Pellegrin, 1912), and *A. ranunculus* Muller, Rapp Py-Daniel & Zuanon, 1994—this fish is most similar in the shape of head and eyes to *A. macrophthalmus* and *A. lithurgicus*. However, the fish collected from the lower Nhamundá had just three branched anal-fin rays, compared to four for both of these species. More individuals will need to be collected, and further investigation of available names in *Ancistrus* carried out in order to discover if this indeed represents an undescribed species.

Two individuals were caught by hand at night from rocky substrates (sampling site NH01). An example of a live specimen is pictured in Fig. 15.

#### Dekeyseria scaphirhyncha

(Kner, 1854)

KP772574

##### Materials

- **Type status:** Other material. **Occurrence:** catalogNumber: 43878; recordedBy: Valéria Nogueira Machado; Emanuell Duarte Ribeiro; Rupert A. Collins; individualCount: 8; otherCatalogNumbers: UFAM:CTGA:14311; UFAM:CTGA:14312; UFAM:CTGA:14313; UFAM:CTGA:14314; UFAM:CTGA:14315; associatedSequences: KP772574; **Taxon:** scientificName: Dekeyseria scaphirhyncha (Kner, 1854); kingdom: Animalia; phylum: Chordata; class: Actinopterygii; order: Siluriformes; family: Loricariidae; genus: Dekeyseria; specificEpithet: scaphirhyncha; scientificNameAuthorship: (Kner, 1854); **Location:** country: Brazil; stateProvince: Pará; locality: Lower Nhamundá River; decimalLatitude: -1.84123; decimalLongitude: -57.07212; geodeticDatum: WGS84; **Identification:** identifiedBy: Rupert A. Collins; **Event:** eventDate: 2013-11; **Record Level:** institutionCode: INPA; basisOfRecord: PreservedSpecimen
- **Type status:** Other material. **Occurrence:** catalogNumber: 43884; recordedBy: Valéria Nogueira Machado; Emanuell Duarte Ribeiro; Rupert A. Collins; individualCount: 1; **Taxon:** scientificName: Dekeyseria scaphirhyncha (Kner, 1854); kingdom: Animalia; phylum: Chordata; class: Actinopterygii; order: Siluriformes; family: Loricariidae; genus: Dekeyseria; specificEpithet: scaphirhyncha; scientificNameAuthorship: (Kner, 1854); **Location:** country: Brazil; stateProvince: Pará; locality: Lower Nhamundá River; decimalLatitude: -1.71782; decimalLongitude: -57.36856; geodeticDatum: WGS84; **Identification:** identifiedBy: Rupert A. Collins; **Event:** eventDate: 2013-11; **Record Level:** institutionCode: INPA; basisOfRecord: PreservedSpecimen

##### Notes

Identification to species level follows Armbruster (2004), Rapp Py-Daniel (1985) and La Monte (1929) based on the following characters: lateral plates with well-developed keels; hypertrophied odontodes present along snout margin; three rows of lateral plates on the caudal peduncle; large interorbital distance; pronounced medial ridge on snout; head plates with sinuous rows of odontodes; interopercular plate with between 15 and 20 strong and distally hooked odontodes; pectoral spine roughly same length as head, with long odontodes; and spots on body roughly same size as those on head.

**Figure 16.**
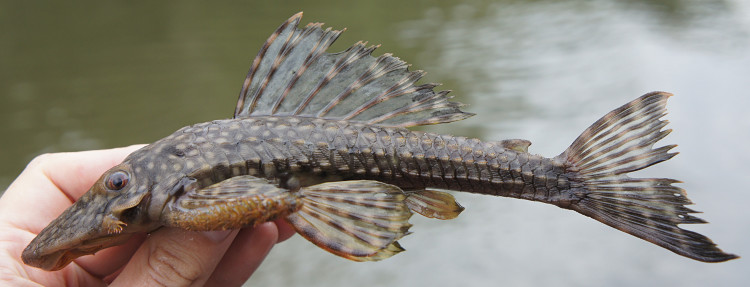
*Dekeyseria scaphirhyncha* live colouration (178.3 mm SL; INPA 43884; UFAM:CTGA:14311).

Nine individuals were caught by hand from woody substrates (sampling sites NH04 and NH08). In addition to the main river stem, the species was also observed in lake and igarapé habitats, and appeared abundant. It was not found in association with rocks. An example of a live specimen is pictured in Fig. 16.

#### Hypancistrus sp. "INPA 43863"

KP772605

##### Materials

- **Type status:** Other material. **Occurrence:** catalogNumber: 43863; recordedBy: Valéria Nogueira Machado; Emanuell Duarte Ribeiro; Rupert A. Collins; individualCount: 12; otherCatalogNumbers: UFAM:CTGA:14552; UFAM:CTGA:14553; UFAM:CTGA:14554; UFAM:CTGA:14555; UFAM:CTGA:14556; associatedSequences: KP772605; **Taxon:** scientificName: Hypancistrus; kingdom: Animalia; phylum: Chordata; class: Actinopterygii; order: Siluriformes; family: Loricariidae; genus: Hypancistrus; vernacularName: sp. "INPA 43863"; taxonRemarks: Possible undescribed species; **Location:** country: Brazil; stateProvince: Pará; locality: Lower Nhamundá River; decimalLatitude: -2.19081; decimalLongitude: -56.7084; geodeticDatum: WGS84; **Identification:** identifiedBy: Rupert A. Collins; **Event:** eventDate: 2013-11; **Record Level:** institutionCode: INPA; basisOfRecord: PreservedSpecimen

##### Notes

Following Armbruster et al. (2007) and Armbruster (2008) we report the following combination of characters allowing a genus-level identification only: five rows of lateral plates on the caudal peduncle; lateral plates not keeled; dentaries forming angle of < 90°; dentary teeth almost twice as long as premaxillary teeth; dentary teeth widely spaced with medial gap between tooth cups as wide as the tooth cups themselves; supraorbital crests very distinct; dark E-shaped pattern on snout (irregular in some individuals); pattern on body of oblique dark bands (almost horizontal wavy stripes in some individuals), with dark bands generally wider than pale bands (i.e. body more dark coloured than pale coloured); dorsal fin with complete bands; and caudal fin with dark vertical bands.

The *Hypancistrus* from the Nhamundá is ostensibly similar to *Hypancistrus furunculus* Armbruster, Lujan & Taphorn (2007), but we hypothesise that it represents a distinct species from *H. furunculus* due to the pronounced supraorbital crests, wide gap between the dentary tooth cups, and colour pattern of wide dark bands and thin pale bands. We await the description of a number of similar species from the Brazilian Shield rivers, some of which may end up being more closely related to this fish than *H. furunculus* is. The species is known in the aquarium trade as *Hypancistrus* sp. "L475" (Konn-Vetterlein and Haagensen 2015)

Twelve individuals were caught by hand at night from rocky substrates (sampling site NH01). Four examples of live specimens are pictured in Fig. 17 to illustrate variation in colour pattern.

#### Hypostomus carinatus

(Steindachner, 1881)

KP772576

KP772577

##### Materials

- **Type status:** Other material. **Occurrence:** catalogNumber: 43879; recordedBy: Valéria Nogueira Machado; Emanuell Duarte Ribeiro; Rupert A. Collins; individualCount: 3; otherCatalogNumbers: UFAM:CTGA:14317; UFAM:CTGA:14318; UFAM:CTGA:14319; associatedSequences: KP772576; KP772577; **Taxon:** scientificName: Hypostomus carinatus (Steindachner, 1881); kingdom: Animalia; phylum: Chordata; class: Actinopterygii; order: Siluriformes; family: Loricariidae; genus: Hypostomus; specificEpithet: carinatus; scientificNameAuthorship: (Steindachner, 1881); **Location:** country: Brazil; stateProvince: Pará; locality: Lower Nhamundá River; decimalLatitude: -1.84123; decimalLongitude: -57.07212; geodeticDatum: WGS84; **Identification:** identifiedBy: Rupert A. Collins; **Event:** eventDate: 2013-11; **Record Level:** institutionCode: INPA; basisOfRecord: PreservedSpecimen
- **Type status:** Other material. **Occurrence:** catalogNumber: 43885; recordedBy: Valéria Nogueira Machado; Emanuell Duarte Ribeiro; Rupert A. Collins; individualCount: 1; otherCatalogNumbers: UFAM:CTGA:14426; **Taxon:** scientificName: Hypostomus carinatus (Steindachner, 1881); kingdom: Animalia; phylum: Chordata; class: Actinopterygii; order: Siluriformes; family: Loricariidae; genus: Hypostomus; specificEpithet: carinatus; scientificNameAuthorship: (Steindachner, 1881); **Location:** country: Brazil; stateProvince: Pará; locality: Lower Nhamundá River; decimalLatitude: -1.71782; decimalLongitude: -57.36856; geodeticDatum: WGS84; **Identification:** identifiedBy: Rupert A. Collins; **Event:** eventDate: 2013-11; **Record Level:** institutionCode: INPA; basisOfRecord: PreservedSpecimen

##### Notes

Identification to species level follows Zawadzki et al. (2013) and Rapp Py-Daniel (1988) based on the following characters: greater than three (around eight to ten) predorsal plates limiting the posterior border of the supraoccipital; elongated caudal peduncle; caudal fin strongly emarginated; dark spots on lighter background; and lower lobe of caudal fin darker than upper lobe.

**Figure 18.**
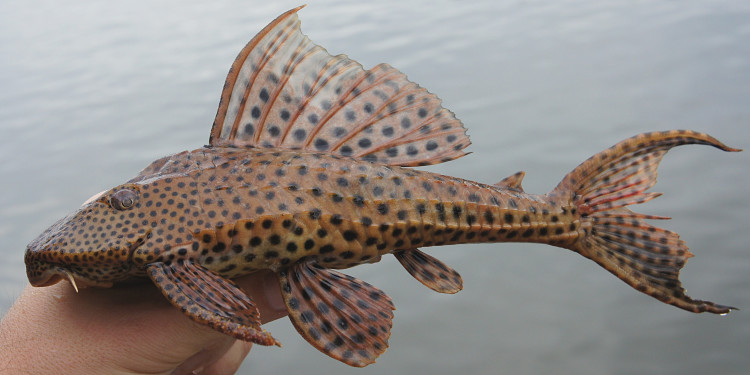
*Hypostomus carinatus* live colouration (196.4 mm SL; INPA 43879; UFAM:CTGA:14317).

Four individuals were caught by hand from woody substrates at the margin of the main river (sampling sites NH04 and NH08). An example of a live specimen is pictured in Fig. 18.

#### Hypostomus macushi

Armbruster & de Souza, 2005

KP772585

##### Materials

- **Type status:** Other material. **Occurrence:** catalogNumber: 46973; recordedBy: Valéria Nogueira Machado; Emanuell Duarte Ribeiro; Rupert A. Collins; individualCount: 1; otherCatalogNumbers: UFAM:CTGA:14425; associatedSequences: KP772585; **Taxon:** scientificName: Hypostomus macushi Armbruster & de Souza, 2005; kingdom: Animalia; phylum: Chordata; class: Actinopterygii; order: Siluriformes; family: Loricariidae; genus: Hypostomus; specificEpithet: macushi; scientificNameAuthorship: Armbruster & de Souza, 2005; **Location:** country: Brazil; stateProvince: Pará; locality: Lower Nhamundá River; decimalLatitude: -1.71782; decimalLongitude: -57.36856; geodeticDatum: WGS84; **Identification:** identifiedBy: Rupert A. Collins; Cláudio H. Zawadzki; **Event:** eventDate: 2013-11; **Record Level:** institutionCode: INPA; basisOfRecord: PreservedSpecimen

##### Notes

Identification to species level follows Armbruster (2003b) and Armbruster and de Souza (2005) based on the following characters: dentaries forming angle of < 80°; spoon-shaped teeth (although not fully formed in this small specimen); widely-spaced large black spots on a light background; and a lack of longitudinal dark stripes.

**Figure 19.**
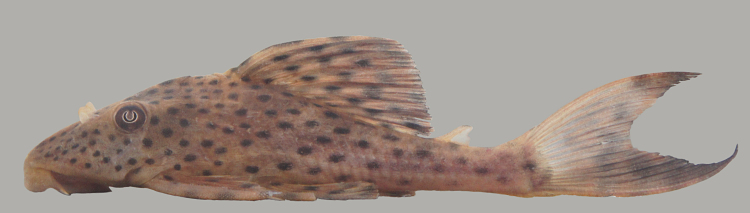
*Hypostomus macushi* live colouration (75.4 mm SL; INPA 46973; UFAM:CTGA:14425).

One individual was caught by hand from woody substrates at the margin of the main river (sampling site NH08). An example of a live specimen is pictured in Fig. 19.

#### Hypostomus plecostomus

(Linnaeus, 1758)

##### Materials

- **Type status:** Other material. **Occurrence:** catalogNumber: 33888; recordedBy: Raphael Leitão; Henrique Lazzarotto; individualCount: 1; **Taxon:** scientificName: Hypostomus plecostomus (Linnaeus, 1758); kingdom: Animalia; phylum: Chordata; class: Actinopterygii; order: Siluriformes; family: Loricariidae; genus: Hypostomus; specificEpithet: plecostomus; scientificNameAuthorship: (Linnaeus, 1758); **Location:** country: Brazil; stateProvince: Pará; locality: Lower Nhamundá River; decimalLatitude: -2.23096; decimalLongitude: -56.77293; geodeticDatum: WGS84; **Identification:** identifiedBy: Cláudio H. Zawadzki; **Event:** eventDate: 2009-09-21; **Record Level:** institutionCode: INPA; basisOfRecord: PreservedSpecimen

##### Notes

Record follows data from a single specimen in the collection of fishes at INPA.

#### Lasiancistrus schomburgkii

(Günther, 1864)

KP772579

##### Materials

- **Type status:** Other material. **Occurrence:** catalogNumber: 43886; recordedBy: Valéria Nogueira Machado; Emanuell Duarte Ribeiro; Rupert A. Collins; individualCount: 1; otherCatalogNumbers: UFAM:CTGA:14329; associatedSequences: KP772579; **Taxon:** scientificName: Lasiancistrus schomburgkii (Günther, 1864); kingdom: Animalia; phylum: Chordata; class: Actinopterygii; order: Siluriformes; family: Loricariidae; genus: Lasiancistrus; specificEpithet: schomburgkii; scientificNameAuthorship: (Günther, 1864); **Location:** country: Brazil; stateProvince: Pará; locality: Lower Nhamundá River; decimalLatitude: -1.6909; decimalLongitude: -57.42231; geodeticDatum: WGS84; **Identification:** identifiedBy: Rupert A. Collins; **Event:** eventDate: 2013-11; **Record Level:** institutionCode: INPA; basisOfRecord: PreservedSpecimen
- **Type status:** Other material. **Occurrence:** catalogNumber: 43892; recordedBy: Valéria Nogueira Machado; Emanuell Duarte Ribeiro; Rupert A. Collins; individualCount: 1; otherCatalogNumbers: UFAM:CTGA:14427; **Taxon:** scientificName: Lasiancistrus schomburgkii (Günther, 1864); kingdom: Animalia; phylum: Chordata; class: Actinopterygii; order: Siluriformes; family: Loricariidae; genus: Lasiancistrus; specificEpithet: schomburgkii; scientificNameAuthorship: (Günther, 1864); **Location:** country: Brazil; stateProvince: Pará; locality: Lower Nhamundá River; decimalLatitude: -1.71782; decimalLongitude: -57.36856; geodeticDatum: WGS84; **Identification:** identifiedBy: Rupert A. Collins; **Event:** eventDate: 2013-11; **Record Level:** institutionCode: INPA; basisOfRecord: PreservedSpecimen

##### Notes

Identification to species level follows Armbruster (2005) based on the following characters: three rows of lateral plates on the caudal peduncle; bar-shaped opercle; snout plates present; lateral plates not keeled; > 30 teeth per jaw ramus; body and fins (except dorsal and caudal) dark with small pale dots; plates not outlined with dark pigment; and caudal fin with darker lower lobe. We note that the whisker-like odontodes characteristic of the genus were not apparent in our juvenile specimens, but we did observe bifurcating tentacules on the interopercular plate, and combined with the other characters, are confident that the specimens belong to *Lasiancistrus schomburgkii* as proposed by Armbruster (2005).

**Figure 20.**
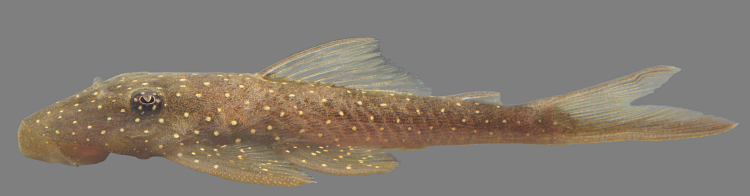
*Lasiancistrus schomburgkii* live colouration (45.2 mm SL; INPA 43886; UFAM:CTGA:14329).

Two individuals were caught by hand from woody substrates at the margin of the main river (sampling sites NH05 and NH08). An example of a live specimen is pictured in Fig. 20.

#### Leporacanthicus galaxias

Isbrücker & Nijssen, 1989

KP772592

##### Materials

- **Type status:** Other material. **Occurrence:** catalogNumber: 43880; recordedBy: Valéria Nogueira Machado; Emanuell Duarte Ribeiro; Rupert A. Collins; individualCount: 3; otherCatalogNumbers: UFAM:CTGA:14328; UFAM:CTGA:14487; UFAM:CTGA:14488; associatedSequences: KP772592; **Taxon:** scientificName: Leporacanthicus galaxias Isbrücker & Nijssen, 1989; kingdom: Animalia; phylum: Chordata; class: Actinopterygii; order: Siluriformes; family: Loricariidae; genus: Leporacanthicus; specificEpithet: galaxias; scientificNameAuthorship: Isbrücker & Nijssen, 1989; **Location:** country: Brazil; stateProvince: Pará; locality: Lower Nhamundá River; decimalLatitude: -1.84123; decimalLongitude: -57.07212; geodeticDatum: WGS84; **Identification:** identifiedBy: Rupert A. Collins; **Event:** eventDate: 2013-11; **Record Level:** institutionCode: INPA; basisOfRecord: PreservedSpecimen

##### Notes

Identification to species level follows Armbruster (2004), Armbruster (2008), Chamon (2007) and Isbrücker and Nijssen (1989) based on the following characters: each premaxilla with three teeth, the inner being very long; lips oval, lacking fimbriae on the upper lip; more than four predorsal plates; tall and narrow supraoccipital crest; and dark body with numerous white dots.

**Figure 21.**
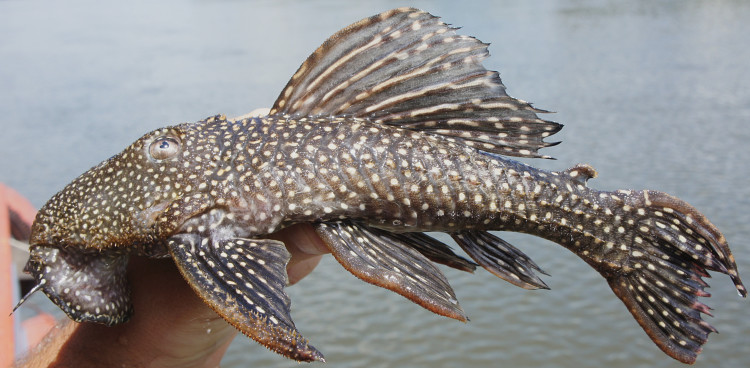
*Leporacanthicus galaxias* live colouration (207.0 mm SL; INPA 43880; UFAM:CTGA:14488)

Three individuals were caught by hand from woody substrates at the margin of the main river (sampling site NH04). An example of a live specimen is pictured in Fig. 21.

#### Peckoltia vittata

(Steindachner, 1881)

KP772575

KP772583

KP772603

##### Materials

- **Type status:** Other material. **Occurrence:** catalogNumber: 43866; recordedBy: Valéria Nogueira Machado; Emanuell Duarte Ribeiro; Rupert A. Collins; individualCount: 1; otherCatalogNumbers: UFAM:CTGA:14305; **Taxon:** scientificName: Peckoltia vittata (Steindachner, 1881); kingdom: Animalia; phylum: Chordata; class: Actinopterygii; order: Siluriformes; family: Loricariidae; genus: Peckoltia; specificEpithet: vittata; scientificNameAuthorship: (Steindachner, 1881); **Location:** country: Brazil; stateProvince: Pará; locality: Lower Nhamundá River; decimalLatitude: -2.17525; decimalLongitude: -56.7115; geodeticDatum: WGS84; **Identification:** identifiedBy: Rupert A. Collins; identificationQualifier: cf. vittata; **Event:** eventDate: 2013-11; **Record Level:** institutionCode: INPA; basisOfRecord: PreservedSpecimen
- **Type status:** Other material. **Occurrence:** catalogNumber: 43881; recordedBy: Valéria Nogueira Machado; Emanuell Duarte Ribeiro; Rupert A. Collins; individualCount: 7; otherCatalogNumbers: UFAM:CTGA:14316; UFAM:CTGA:14325; UFAM:CTGA:14326; UFAM:CTGA:14327; UFAM:CTGA:14494; UFAM:CTGA:14495; UFAM:CTGA:14496; associatedSequences: KP772575; **Taxon:** scientificName: Peckoltia vittata (Steindachner, 1881); kingdom: Animalia; phylum: Chordata; class: Actinopterygii; order: Siluriformes; family: Loricariidae; genus: Peckoltia; specificEpithet: vittata; scientificNameAuthorship: (Steindachner, 1881); **Location:** country: Brazil; stateProvince: Pará; locality: Lower Nhamundá River; decimalLatitude: -1.84123; decimalLongitude: -57.07212; geodeticDatum: WGS84; **Identification:** identifiedBy: Rupert A. Collins; identificationQualifier: cf. vittata; **Event:** eventDate: 2013-11; **Record Level:** institutionCode: INPA; basisOfRecord: PreservedSpecimen
- **Type status:** Other material. **Occurrence:** catalogNumber: 43894; recordedBy: Valéria Nogueira Machado; Emanuell Duarte Ribeiro; Rupert A. Collins; individualCount: 3; otherCatalogNumbers: UFAM:CTGA:14333; UFAM:CTGA:14334; UFAM:CTGA:14335; associatedSequences: KP772583; **Taxon:** scientificName: Peckoltia vittata (Steindachner, 1881); kingdom: Animalia; phylum: Chordata; class: Actinopterygii; order: Siluriformes; family: Loricariidae; genus: Peckoltia; specificEpithet: vittata; scientificNameAuthorship: (Steindachner, 1881); **Location:** country: Brazil; stateProvince: Pará; locality: Lower Nhamundá River; decimalLatitude: -1.6909; decimalLongitude: -57.42231; geodeticDatum: WGS84; **Identification:** identifiedBy: Rupert A. Collins; identificationQualifier: cf. vittata; **Event:** eventDate: 2013-11; **Record Level:** institutionCode: INPA; basisOfRecord: PreservedSpecimen
- **Type status:** Other material. **Occurrence:** catalogNumber: 43887; recordedBy: Valéria Nogueira Machado; Emanuell Duarte Ribeiro; Rupert A. Collins; individualCount: 2; otherCatalogNumbers: UFAM:CTGA:14423; UFAM:CTGA:14424; **Taxon:** scientificName: Peckoltia vittata (Steindachner, 1881); kingdom: Animalia; phylum: Chordata; class: Actinopterygii; order: Siluriformes; family: Loricariidae; genus: Peckoltia; specificEpithet: vittata; scientificNameAuthorship: (Steindachner, 1881); **Location:** country: Brazil; stateProvince: Pará; locality: Lower Nhamundá River; decimalLatitude: -1.71782; decimalLongitude: -57.36856; geodeticDatum: WGS84; **Identification:** identifiedBy: Rupert A. Collins; identificationQualifier: cf. vittata; **Event:** eventDate: 2013-11; **Record Level:** institutionCode: INPA; basisOfRecord: PreservedSpecimen
- **Type status:** Other material. **Occurrence:** catalogNumber: 43869; recordedBy: Valéria Nogueira Machado; Emanuell Duarte Ribeiro; Rupert A. Collins; individualCount: 13; otherCatalogNumbers: UFAM:CTGA:14544; UFAM:CTGA:14545; UFAM:CTGA:14546; associatedSequences: KP772603; **Taxon:** scientificName: Peckoltia vittata (Steindachner, 1881); kingdom: Animalia; phylum: Chordata; class: Actinopterygii; order: Siluriformes; family: Loricariidae; genus: Peckoltia; specificEpithet: vittata; scientificNameAuthorship: (Steindachner, 1881); **Location:** country: Brazil; stateProvince: Pará; locality: Lower Nhamundá River; decimalLatitude: -2.02386; decimalLongitude: -56.78235; geodeticDatum: WGS84; **Identification:** identifiedBy: Rupert A. Collins; identificationQualifier: cf. vittata; **Event:** eventDate: 2013-11; **Record Level:** institutionCode: INPA; basisOfRecord: PreservedSpecimen
- **Type status:** Other material. **Occurrence:** catalogNumber: 43864; recordedBy: Valéria Nogueira Machado; Emanuell Duarte Ribeiro; Rupert A. Collins; individualCount: 2; otherCatalogNumbers: UFAM:CTGA:14550; UFAM:CTGA:14551; **Taxon:** scientificName: Peckoltia vittata (Steindachner, 1881); kingdom: Animalia; phylum: Chordata; class: Actinopterygii; order: Siluriformes; family: Loricariidae; genus: Peckoltia; specificEpithet: vittata; scientificNameAuthorship: (Steindachner, 1881); **Location:** country: Brazil; stateProvince: Pará; locality: Lower Nhamundá River; decimalLatitude: -2.19081; decimalLongitude: -56.7084; geodeticDatum: WGS84; **Identification:** identifiedBy: Rupert A. Collins; identificationQualifier: cf. vittata; **Event:** eventDate: 2013-11; **Record Level:** institutionCode: INPA; basisOfRecord: PreservedSpecimen
- **Type status:** Other material. **Occurrence:** catalogNumber: 33866; recordedBy: Raphael Leitão; Henrique Lazzarotto; individualCount: 2; **Taxon:** scientificName: Peckoltia vittata (Steindachner, 1881); kingdom: Animalia; phylum: Chordata; class: Actinopterygii; order: Siluriformes; family: Loricariidae; genus: Peckoltia; specificEpithet: vittata; scientificNameAuthorship: (Steindachner, 1881); **Location:** country: Brazil; stateProvince: Pará; locality: Lower Nhamundá River; decimalLatitude: -2.19056; decimalLongitude: -56.71167; geodeticDatum: WGS84; **Identification:** identifiedBy: R. Frederico; Rupert A. Collins; identificationQualifier: cf. vittata; **Event:** eventDate: 2009-09-21; **Record Level:** institutionCode: INPA; basisOfRecord: PreservedSpecimen

##### Notes

Identification to species level follows Armbruster (2008) based on the following characters: evertible cheek plates with > 10 hypertrophied odontodes; dentaries forming angle of < 90°; deep body, not dorso-ventrally flattened; lips lacking fimbriae; villiform teeth of equal size in premaxilla and dentary; colour pattern of four irregular dorsal saddles (first and second are usually combined in small specimens); dorsal and caudal fins without orange margin (in life); head plates not outlined in black; dark bar between eyes, e-shaped dark blotch on the snout (broken in some specimens); and fins with dark and light bands of approximately equal width (irregular in some specimens).

An interesting observation is that our specimens exhibited variation in terms of the degree of abdomen plating and markings: adult specimens from collection points near the mouth of the river (sampling sites NH01, NH02 and NH12) showed complete abdominal plating and a vermiculated pattern (Fig. 22a, b); those from further up the river (sampling sites NH04, NH05 and NH08) lacked abdominal plating and associated colouration (Fig. 22c, d), with the exception of one individual that was partially plated with discrete spots (Fig. 22e, f). In light of this variation we were hesitant in referring all these individuals to *Peckoltia vittata*, and hence we use the "*cf.*" qualifier. The three DNA barcodes we generated from the different phenotypes/sites, however, were identical.

Twenty-eight individuals were caught by hand from both woody substrates (sampling sites NH02, NH04, NH05, and NH08) and rocky substrates (sampling sites NH01 and NH12). Two further specimens of this species are reported from the collection of fishes at INPA.

#### Pseudolithoxus sp. "INPA 43888"

KP772584

KP772590

KP772591

##### Materials

- **Type status:** Other material. **Occurrence:** catalogNumber: INPA 43889; INPA 43888; recordedBy: Valéria Nogueira Machado; Emanuell Duarte Ribeiro; Rupert A. Collins; individualCount: 17; otherCatalogNumbers: UFAM:CTGA:14422; UFAM:CTGA:14482; UFAM:CTGA:14483; UFAM:CTGA:14484; UFAM:CTGA:14485; UFAM:CTGA:14486; associatedSequences: KP772584; KP772590; KP772591; **Taxon:** scientificName: Pseudolithoxus; kingdom: Animalia; phylum: Chordata; class: Actinopterygii; order: Siluriformes; family: Loricariidae; genus: Pseudolithoxus; vernacularName: sp. "INPA 43888"; taxonRemarks: Undescribed species; **Location:** country: Brazil; stateProvince: Pará; locality: Lower Nhamundá River; decimalLatitude: -1.71782; decimalLongitude: -57.36856; geodeticDatum: WGS84; **Identification:** identifiedBy: Rupert A. Collins; **Event:** eventDate: 2013-11; **Record Level:** institutionCode: INPA; basisOfRecord: PreservedSpecimen

##### Notes

Identification to genus level follows Armbruster and Provenzano (2000) and Lujan and Birindelli (2011) based on the following characters: three rows of lateral plates on the caudal peduncle; lateral plates without pronounced keels; fully plated snout lacking tentacles; hypertrophied odontodes on the snout in both females and males; lack of whisker-like odontodes on the cheek plates; extremely hypertrophied odontodes on an elongated pectoral-fin spine in both females and males; sucking disk without fimbriae; and greater than 20 teeth per jaw ramus.

Using the above references we were unable to identify the specimens to species level, as the individuals differed from the colour patterns diagnostic of the other species in the genus. We hypothesise that these individuals represent a new species of *Pseudolithoxus*, and intend to document this in more detail in a separate publication.

**Figure 23.**
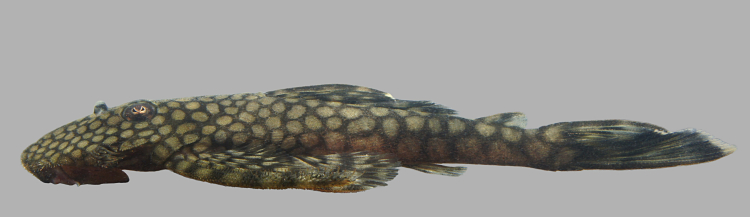
*Pseudolithoxus* sp. "INPA 43888" live colouration of juvenile (72.6 mm SL; INPA 43889; UFAM:CTGA:14486).

Seventeen individuals were captured by hand from crevices in rocky habitats exposed to strong current (sampling site NH08). Live colouration is shown in Fig. 23.

#### Loricariinae

#### Farlowella nattereri

Steindachner, 1910

KP772581

##### Materials

- **Type status:** Other material. **Occurrence:** catalogNumber: 43891; recordedBy: Valéria Nogueira Machado; Emanuell Duarte Ribeiro; Rupert A. Collins; individualCount: 1; otherCatalogNumbers: UFAM:CTGA:14331; associatedSequences: KP772581; **Taxon:** scientificName: Farlowella nattereri Steindachner, 1910; kingdom: Animalia; phylum: Chordata; class: Actinopterygii; order: Siluriformes; family: Loricariidae; genus: Farlowella; specificEpithet: nattereri; scientificNameAuthorship: Steindachner, 1910; **Location:** country: Brazil; stateProvince: Pará; locality: Lower Nhamundá River; decimalLatitude: -1.6909; decimalLongitude: -57.42231; geodeticDatum: WGS84; **Identification:** identifiedBy: Rupert A. Collins; **Event:** eventDate: 2013-11; **Record Level:** institutionCode: INPA; basisOfRecord: PreservedSpecimen

##### Notes

Identification to species level follows Retzer and Page (1997) and Retzer (2006) based on the following characters: seven predorsal plates; dorsal fin located opposite anal fin; three rows of abdominal plates; five rows of anterior lateral plates, with middle row incomplete; plates of second lateral row diamond-shaped; fourth row of anterior lateral plates sharply keeled; odontodes on lateral plates small; snout-mouth-length / head-length > 0.5; body-depth / pelvic-fin-length < 0.86; pectoral-fin-length / snout-mouth-length > 0.65; snout-mouth-length / pectoral-fin-length > 1.0; and fin spines and rays with dark spots.

The above characters are consistent with *F. nattereri*, but some key differences in colour pattern are noted. Retzer and Page (1997) report: for most specimens of *F. nattereri*, the first anal and dorsal fin rays are entirely darkly pigmented (our specimen has spotted rays); a distinct dorso-lateral dark-stripe is present from base of snout to dorsal fin (this stripe was not apparent in the preserved specimen, but was observed in life); and upper and lower caudal fin lobes pigmented with dark stripes of equal size, with stripes often not reaching caudal fin base (the stripes in our specimen reached the caudal base). Retzer and Page (1997) recognise that *F. nattereri* probably comprises a complex of species.

**Figure 24.**
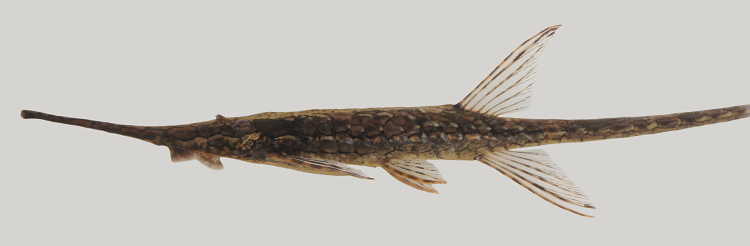
*Farlowella nattereri* live colouration (199.8 mm SL; INPA 43891; UFAM:CTGA:14331).

**Figure 25.**
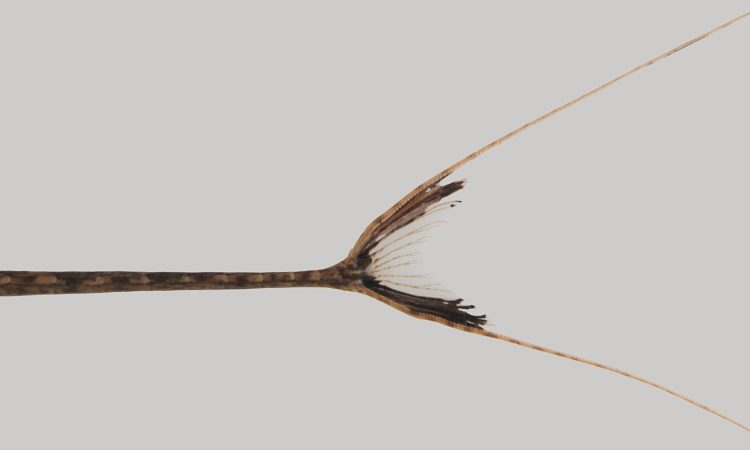
*Farlowella nattereri* caudal fin (INPA 43891; UFAM:CTGA:14331).

One individual was caught by hand from shallow, fast flowing water over a rocky/sandy substrate (sampling site NH05). The live specimen is pictured in Fig. 24 (caudal fin in Fig. 25)

#### Limatulichthys griseus

(Eigenmann, 1909)

##### Materials

- **Type status:** Other material. **Occurrence:** catalogNumber: 33892; recordedBy: Raphael Leitão; Henrique Lazzarotto; individualCount: 1; **Taxon:** scientificName: Limatulichthys griseus (Eigenmann, 1909); kingdom: Animalia; phylum: Chordata; class: Actinopterygii; order: Siluriformes; family: Loricariidae; genus: Limatulichthys; specificEpithet: griseus; scientificNameAuthorship: (Eigenmann, 1909); **Location:** country: Brazil; stateProvince: Pará; locality: Lower Nhamundá River; decimalLatitude: -2.19972; decimalLongitude: -56.69222; geodeticDatum: WGS84; **Identification:** identifiedBy: Rupert A. Collins; **Event:** eventDate: 2009-09-23; **Record Level:** institutionCode: INPA; basisOfRecord: PreservedSpecimen

##### Notes

Record follows data from a single specimen in the collection of fishes at INPA. The specimen had been identified there as *Limatulichthys* sp., but we refer to the fish to *L. griseus* as reported by Londoño-Burbano et al. (2014), as it does not disagree with their concept of that species.

#### Loricaria cataphracta

Linnaeus, 1758

KP772582

##### Materials

- **Type status:** Other material. **Occurrence:** catalogNumber: 43893; recordedBy: Valéria Nogueira Machado; Emanuell Duarte Ribeiro; Rupert A. Collins; individualCount: 1; otherCatalogNumbers: UFAM:CTGA:14332; associatedSequences: KP772582; **Taxon:** scientificName: Loricaria cataphracta Linnaeus, 1758; kingdom: Animalia; phylum: Chordata; class: Actinopterygii; order: Siluriformes; family: Loricariidae; genus: Loricaria; specificEpithet: cataphracta; scientificNameAuthorship: Linnaeus, 1758; **Location:** country: Brazil; stateProvince: Pará; locality: Lower Nhamundá River; decimalLatitude: -1.6909; decimalLongitude: -57.42231; geodeticDatum: WGS84; **Identification:** identifiedBy: Rupert A. Collins; **Event:** eventDate: 2013-11; **Record Level:** institutionCode: INPA; basisOfRecord: PreservedSpecimen

##### Notes

Identification to species level follows Isbrücker (1981), Thomas and Rapp Py-Daniel (2008) and Thomas and Sabaj Pérez (2010) based on the following characters: elongate lip filaments; three premaxillary teeth per ramus; premaxillary teeth approximately twice as long as dentary teeth; developed odontode crests on head and dorsal trunk plates; 34 lateral plates; 19 coalesced lateral plates; dorsal fin spine not elongated (24% of SL); post-orbital notch relatively well developed; abdomen mostly plated (with the exception of an anterior v-shaped naked area over the pectoral girdle); and all fins except anal fin with dark sub-distal bands (most prominent on caudal and dorsal fins). Identification of this species is tentative, as there appears to be considerable variation in the *L. cataphracta* group. We await the forthcoming systematic revision of the genus (as mentioned in Thomas and Sabaj Pérez 2010).

**Figure 26.**
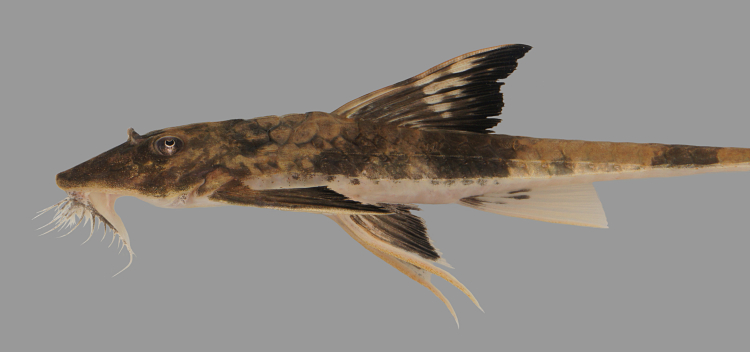
*Loricaria cataphracta* live colouration (144.3 mm SL; INPA 43893; UFAM:CTGA:14332). Image flipped horizontally.

One individual was caught by hand from shallow, fast flowing water over a rocky/sandy substrate (sampling site NH05). The live specimen is pictured in Fig. 26.

#### Pseudoloricaria laeviuscula

(Valenciennes, 1840)

KP772602

##### Materials

- **Type status:** Other material. **Occurrence:** catalogNumber: 43870; recordedBy: Valéria Nogueira Machado; Emanuell Duarte Ribeiro; Rupert A. Collins; individualCount: 3; otherCatalogNumbers: UFAM:CTGA:14541; UFAM:CTGA:14542; UFAM:CTGA:14543; associatedSequences: KP772602; **Taxon:** scientificName: Pseudoloricaria laeviuscula (Valenciennes, 1840); kingdom: Animalia; phylum: Chordata; class: Actinopterygii; order: Siluriformes; family: Loricariidae; genus: Pseudoloricaria; specificEpithet: laeviuscula; scientificNameAuthorship: (Valenciennes, 1840); **Location:** country: Brazil; stateProvince: Pará; locality: Lower Nhamundá River; decimalLatitude: -2.02386; decimalLongitude: -56.78235; geodeticDatum: WGS84; **Identification:** identifiedBy: Rupert A. Collins; **Event:** eventDate: 2013-11; **Record Level:** institutionCode: INPA; basisOfRecord: PreservedSpecimen
- **Type status:** Other material. **Occurrence:** catalogNumber: 43895; recordedBy: Valéria Nogueira Machado; Emanuell Duarte Ribeiro; Rupert A. Collins; individualCount: 4; otherCatalogNumbers: UFAM:CTGA:14042; UFAM:CTGA:14043; UFAM:CTGA:14046; UFAM:CTGA:14047; **Taxon:** scientificName: Pseudoloricaria laeviuscula (Valenciennes, 1840); kingdom: Animalia; phylum: Chordata; class: Actinopterygii; order: Siluriformes; family: Loricariidae; genus: Pseudoloricaria; specificEpithet: laeviuscula; scientificNameAuthorship: (Valenciennes, 1840); **Location:** country: Brazil; stateProvince: Pará; locality: Lower Nhamundá River; decimalLatitude: -1.6909; decimalLongitude: -57.42231; geodeticDatum: WGS84; **Identification:** identifiedBy: Rupert A. Collins; **Event:** eventDate: 2013-11; **Record Level:** institutionCode: INPA; basisOfRecord: PreservedSpecimen
- **Type status:** Other material. **Occurrence:** catalogNumber: 33893; recordedBy: Raphael Leitão; Henrique Lazzarotto; individualCount: 1; **Taxon:** scientificName: Pseudoloricaria laeviuscula (Valenciennes, 1840); kingdom: Animalia; phylum: Chordata; class: Actinopterygii; order: Siluriformes; family: Loricariidae; genus: Pseudoloricaria; specificEpithet: laeviuscula; scientificNameAuthorship: (Valenciennes, 1840); **Location:** country: Brazil; stateProvince: Pará; locality: Lower Nhamundá River; decimalLatitude: -1.99972; decimalLongitude: -56.51611; geodeticDatum: WGS84; **Identification:** identifiedBy: W. Ohara; Rupert A. Collins; identificationQualifier: aff. laeviuscula; **Event:** eventDate: 2009-09-23; **Record Level:** institutionCode: INPA; basisOfRecord: PreservedSpecimen

##### Notes

Identification to species level follows Isbrücker and Nijssen (1976) and Covain and Fisch-Muller (2007) based on the following characters: lower lip bilobate with median furrow; whip on upper caudal spine absent; abdomen covered with small plates lacking organisation; elliptical area of abdominal plates at level of pelvic girdle absent; rostrum not strongly pronounced; pelvic-fin spine longer than last pelvic-fin branched ray; colouration comprising dark dots (except ventral surface and anal fin); lower lobe of caudal darker than upper; and basicaudal spot present in juveniles.

**Figure 27.**
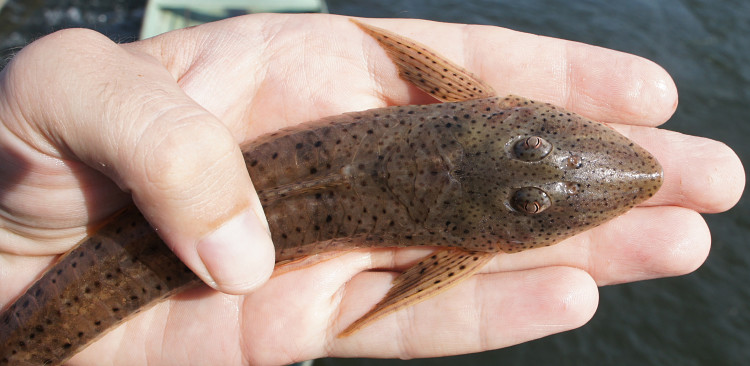
*Pseudoloricaria laeviuscula* live colouration (216.4 mm SL; INPA 43870; UFAM:CTGA:14542).

Three adult individuals were caught by hand-net at night over a sandy/silty substrate (sampling site NH12), and four juveniles were caught further upstream on the sandy margins of the river (sampling site NH05). An example of a live adult specimen is pictured in Fig. 27.

One further specimen record of this species was obtained from the fish collection at INPA; this individual had been identified as P. aff. laeviuscula, but we include it here under *P. laeviuscula* until further information becomes available.

#### Rineloricaria lanceolata

(Günther, 1868)

KP772580

##### Materials

- **Type status:** Other material. **Occurrence:** catalogNumber: 43896; recordedBy: Valéria Nogueira Machado; Emanuell Duarte Ribeiro; Rupert A. Collins; individualCount: 2; otherCatalogNumbers: UFAM:CTGA:14330; UFAM:CTGA:14044; associatedSequences: KP772580; **Taxon:** scientificName: Rineloricaria lanceolata (Günther, 1868); kingdom: Animalia; phylum: Chordata; class: Actinopterygii; order: Siluriformes; family: Loricariidae; genus: Rineloricaria; specificEpithet: lanceolata; scientificNameAuthorship: (Günther, 1868); **Location:** country: Brazil; stateProvince: Pará; locality: Lower Nhamundá River; decimalLatitude: -1.6909; decimalLongitude: -57.42231; geodeticDatum: WGS84; **Identification:** identifiedBy: Rupert A. Collins; **Event:** eventDate: 2013-11; **Record Level:** institutionCode: INPA; basisOfRecord: PreservedSpecimen

##### Notes

Identification to species level follows Vera-Alcaraz et al. (2012) and Fichberg and Chamon (2008) based on the following characters: postorbital notch present; inferior lip with short, round papillae; teeth on dentary larger than premaxilla; four rows of lateral plates; all fins with a broad longitudinal dark band parallel to the first rays (fins almost entirely dark in our specimen); lower lip margin with long fringes; and dorsal surface of head and predorsal region with two longitudinal dark bands. Note that the characteristic dorsal breeding odontodes of *R. lanceolata* were not visible in this single specimen (probably female).

**Figure 28.**
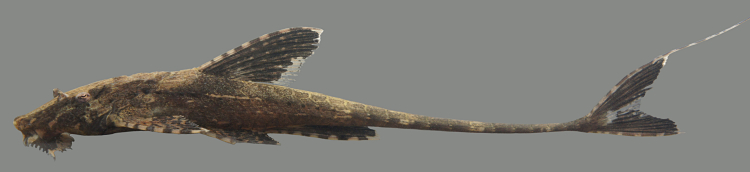
*Rineloricaria lanceolata* live colouration (86.8 mm SL; INPA 43896; UFAM:CTGA:14330).

Two individuals were caught by hand from shallow, fast flowing water over a rocky/sandy substrate on the main river (sampling site NH05). A live specimen is pictured in Fig. 28.

#### Pimelodidae

#### Calophysus macropterus

(Lichtenstein, 1819)

KP772598

##### Materials

- **Type status:** Other material. **Occurrence:** catalogNumber: 43898; recordedBy: Valéria Nogueira Machado; Emanuell Duarte Ribeiro; Rupert A. Collins; individualCount: 1; otherCatalogNumbers: UFAM:CTGA:14408; **Taxon:** scientificName: Calophysus macropterus (Lichtenstein, 1819); kingdom: Animalia; phylum: Chordata; class: Actinopterygii; order: Siluriformes; family: Pimelodidae; genus: Calophysus; specificEpithet: macropterus; scientificNameAuthorship: (Lichtenstein, 1819); **Location:** country: Brazil; stateProvince: Pará; locality: Lower Nhamundá River; decimalLatitude: -1.67511; decimalLongitude: -57.47678; geodeticDatum: WGS84; **Identification:** identifiedBy: Rupert A. Collins; **Event:** eventDate: 2013-11; **Record Level:** institutionCode: INPA; basisOfRecord: PreservedSpecimen
- **Type status:** Other material. **Occurrence:** catalogNumber: 43871; recordedBy: Valéria Nogueira Machado; Emanuell Duarte Ribeiro; Rupert A. Collins; individualCount: 4; otherCatalogNumbers: UFAM:CTGA:14528; UFAM:CTGA:14529; UFAM:CTGA:14530; UFAM:CTGA:14531; associatedSequences: KP772598; **Taxon:** scientificName: Calophysus macropterus (Lichtenstein, 1819); kingdom: Animalia; phylum: Chordata; class: Actinopterygii; order: Siluriformes; family: Pimelodidae; genus: Calophysus; specificEpithet: macropterus; scientificNameAuthorship: (Lichtenstein, 1819); **Location:** country: Brazil; stateProvince: Pará; locality: Lower Nhamundá River; decimalLatitude: -2.02386; decimalLongitude: -56.78235; geodeticDatum: WGS84; **Identification:** identifiedBy: Rupert A. Collins; **Event:** eventDate: 2013-11; **Record Level:** institutionCode: INPA; basisOfRecord: PreservedSpecimen

##### Notes

Identification to species level follows Eigenmann and Eigenmann (1890) based on the following characters: two rows of maxillary teeth (posterior row very small and hidden within the skin folds) and one row of dentary teeth; first dorsal and pectoral rays not spinous; adipose fin long; barbels flattened; upper jaw slightly prognathous; and dark spots on flanks and adipose fin.

**Figure 29.**
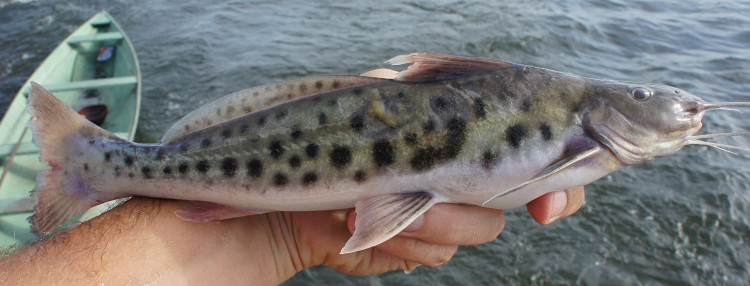
*Calophysus macropterus* live colouration (254.0 mm SL; INPA 43871; UFAM:CTGA:14531).

Five individuals were caught after being attracted to the boat by suspending a dead-fish bait in the water (sampling site NH12). An example of a live specimen is pictured in Fig. 29.

#### Phractocephalus hemioliopterus

(Bloch & Schneider, 1801)

KP772589

##### Materials

- **Type status:** Other material. **Occurrence:** catalogNumber: 14459; recordedBy: Valéria Nogueira Machado; Emanuell Duarte Ribeiro; Rupert A. Collins; individualCount: 1; associatedSequences: KP772589; **Taxon:** scientificName: Phractocephalus hemioliopterus (Bloch & Schneider, 1801); kingdom: Animalia; phylum: Chordata; class: Actinopterygii; order: Siluriformes; family: Pimelodidae; genus: Phractocephalus; specificEpithet: hemioliopterus; scientificNameAuthorship: (Bloch & Schneider, 1801); **Location:** country: Brazil; stateProvince: Pará; locality: Lower Nhamundá River; decimalLatitude: -1.67511; decimalLongitude: -57.47678; geodeticDatum: WGS84; **Identification:** identifiedBy: Rupert A. Collins; **Event:** eventDate: 2013-11; **Record Level:** institutionCode: UFAM; collectionCode: CTGA; basisOfRecord: PreservedSpecimen

##### Notes

Identification to species level follows Lundberg and Aguilera (2003) and Mol (2012) based on the following characters: dermal bones of the skull coarsely sculpted with reticulated ridges surrounding rounded pits; supraoccipital process greatly expanded laterally; anterior nuchal plate enlarged; colour pattern with dark upper and white/yellow lower parts of flank; and caudal fin bright red/orange.

**Figure 30.**
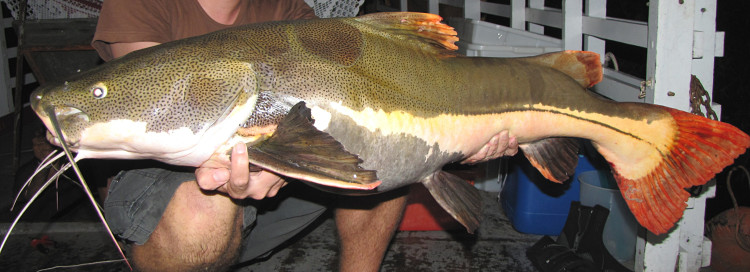
*Phractocephalus hemioliopterus* live colouration (approx 100 cm SL; UFAM:CTGA:14459). Voucher not retained.

One individual was caught by baited hand-line in a deep pool of the main river. The live specimen is pictured in Fig. 30.

#### Pimelodus blochii

Valenciennes, 1840

##### Materials

- **Type status:** Other material. **Occurrence:** catalogNumber: 33886; recordedBy: Raphael Leitão; Henrique Lazzarotto; individualCount: 1; **Taxon:** scientificName: Pimelodus blochii Valenciennes, 1840; kingdom: Animalia; phylum: Chordata; class: Actinopterygii; order: Siluriformes; family: Pimelodidae; genus: Pimelodus; specificEpithet: blochii; scientificNameAuthorship: Valenciennes, 1840; **Location:** country: Brazil; stateProvince: Pará; locality: Lower Nhamundá River; decimalLatitude: -2.23083; decimalLongitude: -56.77306; geodeticDatum: WGS84; **Identification:** identifiedBy: R. Frederico; **Event:** eventDate: 2009-09-21; **Record Level:** institutionCode: INPA; basisOfRecord: PreservedSpecimen

##### Notes

Record follows data from a single specimen in the collection of fishes at INPA.

#### Pinirampus pirinampu

(Spix & Agassiz, 1829)

KP772587

##### Materials

- **Type status:** Other material. **Occurrence:** catalogNumber: 14443; recordedBy: Valéria Nogueira Machado; Emanuell Duarte Ribeiro; Rupert A. Collins; individualCount: 1; associatedSequences: KP772587; **Taxon:** scientificName: Pinirampus pirinampu (Spix & Agassiz, 1829); kingdom: Animalia; phylum: Chordata; class: Actinopterygii; order: Siluriformes; family: Pimelodidae; genus: Pinirampus; specificEpithet: pirinampu; scientificNameAuthorship: (Spix & Agassiz, 1829); **Location:** country: Brazil; stateProvince: Pará; locality: Lower Nhamundá River; decimalLatitude: -1.67511; decimalLongitude: -57.47678; geodeticDatum: WGS84; **Identification:** identifiedBy: Valéria Nogueira Machado; **Event:** eventDate: 2013-11; **Record Level:** institutionCode: UFAM; collectionCode: CTGA; basisOfRecord: PreservedSpecimen

##### Notes

Identification to species level follows Eigenmann and Eigenmann (1890) based on the following characters: adipose fin long; top of head covered with skin; upper jaw slightly prognathous; and barbels broad and long with broad membranaceous border on the posterior margins.

One individual was caught by gill net, but was not vouchered or photographed.

#### Pseudoplatystoma reticulatum

Eigenmann & Eigenmann, 1889

KP772588

##### Materials

- **Type status:** Other material. **Occurrence:** catalogNumber: 14451; 14452; 14453; 14454; recordedBy: Valéria Nogueira Machado; Emanuell Duarte Ribeiro; Rupert A. Collins; individualCount: 4; associatedSequences: KP772588; **Taxon:** scientificName: Pseudoplatystoma reticulatum Eigenmann & Eigenmann, 1889; kingdom: Animalia; phylum: Chordata; class: Actinopterygii; order: Siluriformes; family: Pimelodidae; genus: Pseudoplatystoma; specificEpithet: reticulatum; scientificNameAuthorship: Eigenmann & Eigenmann, 1889; **Location:** country: Brazil; stateProvince: Pará; locality: Lower Nhamundá River; decimalLatitude: -1.67511; decimalLongitude: -57.47678; geodeticDatum: WGS84; **Identification:** identifiedBy: Rupert A. Collins; **Event:** eventDate: 2013-11; **Record Level:** institutionCode: UFAM; collectionCode: CTGA; basisOfRecord: PreservedSpecimen

##### Notes

Identification to species level follows Buitrago-Suárez and Burr (2007) based on the following characters: head strongly depressed with extended cranial fontanelle; loop-like dark bars forming reticulated pattern which extends far below lateral line and connects dorsally; no clear demarcation between dark dorsal and pale ventral regions; and caudal fin with fewer than 45 spots.

While we follow the taxonomy of Buitrago-Suárez and Burr (2007), we also consider the possibility that *P. reticulatum* Eigenmann & Eigenmann, 1889 is a junior subjective synonym of *P. fasciatum* (Linnaeus, 1766) in light of the study of Carvalho-Costa et al. (2011). These authors reported minimal genetic differentiation among the taxa considered conspecific with *P. fasciatum* previous to the study of Buitrago-Suárez and Burr (2007).

**Figure 31.**
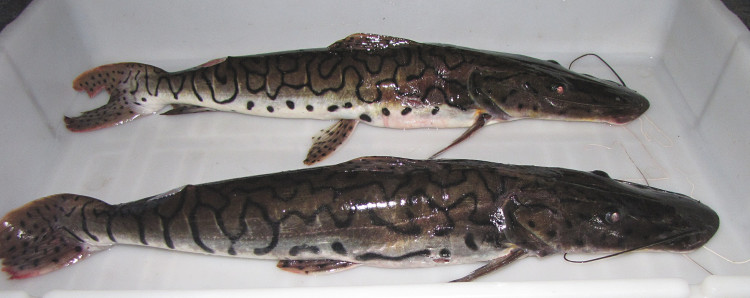
*Pseudoplatystoma reticulatum* live colouration (front specimen 46 cm SL). Vouchers not retained.

Four individuals were caught at night using gill nets in a lake connected to the river. An example of two live specimens is pictured in Fig. 31.

#### Pseudopimelodidae

#### Batrochoglanis villosus

(Eigenmann, 1912)

KP772594

##### Materials

- **Type status:** Other material. **Occurrence:** catalogNumber: 43882; recordedBy: Valéria Nogueira Machado; Emanuell Duarte Ribeiro; Rupert A. Collins; individualCount: 4; otherCatalogNumbers: UFAM:CTGA:14497; UFAM:CTGA:14498; UFAM:CTGA:14499; UFAM:CTGA:14500; associatedSequences: KP772594; **Taxon:** scientificName: Batrochoglanis villosus (Eigenmann, 1912); kingdom: Animalia; phylum: Chordata; class: Actinopterygii; order: Siluriformes; family: Pseudopimelodidae; genus: Batrochoglanis; specificEpithet: villosus; scientificNameAuthorship: (Eigenmann, 1912); **Location:** country: Brazil; stateProvince: Pará; locality: Lower Nhamundá River; decimalLatitude: -1.84123; decimalLongitude: -57.07212; geodeticDatum: WGS84; **Identification:** identifiedBy: Rupert A. Collins; **Event:** eventDate: 2013-11; **Record Level:** institutionCode: INPA; basisOfRecord: PreservedSpecimen

##### Notes

Identification to species level follows Shibatta and Pavanelli (2005), Mees (1974) and Eigenmann (1912) based on the following characters: lower jaw not projecting beyond upper jaw; head large and rounded in dorsal view; head and body with numerous small papillae; insertion of pelvic fin though vertical of posterior base of dorsal; short caudal peduncle; rounded caudal fin; post-cleithral process short, not reaching vertical through dorsal fin origin; axillary pore absent; lateral-line canal terminating on caudal peduncle; premaxillary dentigerous plates with lateral margins posteriorly prolonged; colour brown, with dark mottled appearance; and caudal fin with dark dots irregularly distributed.

An important inconsistency should be noted regarding the caudal fin shape. Eigenmann (1912) described the caudal fin of *B. villosus* as being "notched", but figured a specimen with a rounded caudal fin (Mees 1974). The photograph of the holotype (FMNH 53219) on the All Species Catfish Inventory Web page (http://acsi.acnatsci.org/base/image_list.html?mode=genus&genus=Pseudopimelodus) shows a fish lacking most of the caudal fin. The specimens we collected had a rounded caudal fin.

**Figure 32.**
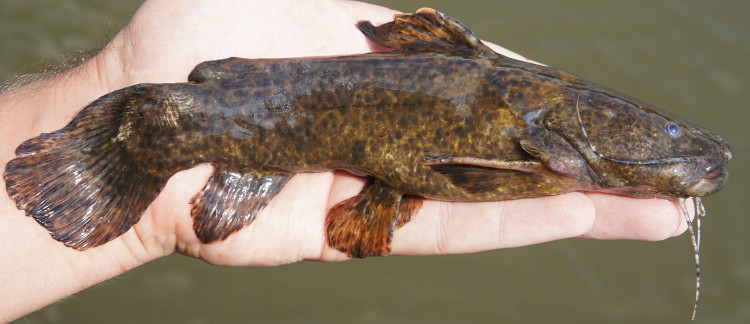
*Batrochoglanis villosus* live colouration (176.0 mm SL; INPA 43882; UFAM:CTGA:14497).

Four individuals were caught by hand from their lodgements in woody substrates at the margin of the main river (sampling site NH04). An example of a live specimen is pictured in Fig. 32.

#### Trichomycteridae

#### Pygidianops amphioxus

de Pinna & Kirovsky, 2011

##### Notes

Record follows data from de Pinna and Kirovsky (2011).

## Analysis

### Desk survey

Our survey of the online databases revealed a single species record from the Rio Nhamundá: an unidentified *Ossancora* (Doradidae) which on further investigation was found to comprise two paratype lots of *Ossancora asterophysa* Birindelli & Sabaj Pérez, 2011 (ROM 88244, MZUSP 7838). The literature survey revealed three further species from the river: *Hassar orestis* (Steindachner, 1875) (MZUSP 9547); *Pygidianops amphioxus* de Pinna & Kirovsky, 2011 (MZUSP 104675); and *Centromochlus* sp. (INPA 35087). By contrast, the survey of the Web databases and checklists for the Trombetas river listed 44 siluriform species, while the study of Ferreira (1993) listed 95 siluriform species from the Trombetas. From the Uatumã river, the desk survey of Web databases and checklists provided five species.

The collection of fishes at INPA held nine lots of catfishes previously collected from the Nhamundá. One of these records (*Centromochlus* sp., INPA 35087) was also recorded in the literature survey (Sarmento-Soares et al. 2013). As stated previously, because this lot was out on loan at the time of writing this manuscript, we were not able to examine the specimens and have therefore omitted this species from the total species count due its ambiguity.

Our review of the genetic databases shows GenBank holds COI data for nine of the 24 species we obtained DNA barcodes from and identified as a known species (number of records in parentheses): *Trachycorystes trachycorystes* (1), *Astrodoras asterifrons* (1), *Scorpiodoras heckelii* (2), *Farlowella nattereri* (1), *Peckoltia vittata* (1), *Phractocephalus hemioliopterus* (1), *Pinirampus pirinampu* (7), *Pseudoplatystoma reticulatum* (12), and *Batrochoglanis villosus* (1). The BOLD database also holds COI data for nine species: *Farlowella nattereri* (3), *Loricaria cataphracta* (5), *Peckoltia vittata* (16), *Pseudoloricaria laeviuscula* (3), *Rineloricaria lanceolata* (3), *Phractocephalus hemioliopterus* (4), *Pinirampus pirinampu* (10), *Pseudoplatystoma reticulatum* (35), and *Batrochoglanis villosus* (1).

In addition to the species listed above and available in GenBank, we provide COI data for 17 putative species with no current sequences apparently available in either GenBank or BOLD: *Auchenipterichthys longimanus*, *Tatia musaica*, T. aff. musaica, *T. nigra*, *Goeldiella eques*, *Pimelodella* sp., *Ancistrus dolichopterus*, *Ancistrus* sp. "INPA 43862", *Dekeyseria scaphirhyncha*, *Hypancistrus* sp. "INPA 43863", *Hypoptopoma incognitum*, *Hypostomus carinatus*, *H. macushi*, *Lasiancistrus schomburgkii*, *Leporacanthicus galaxias*, *Pseudolithoxus* sp. "INPA 43888", and *Calophysus macropterus*.

## Discussion

### Inventory of Siluriformes

Our desk and museum collection surveys show that few ichthyological surveys of the Rio Nhamundá have taken place, and that our collection is one of the first to be made on the river. Not including the unverified *Centromochlus* sp., we report three species from the desk survey, eight species from the museum survey, and 28 species from our field survey; three species (Tatia aff. musaica, Peckoltia cf. vittata, *Pseudoloricaria laeviuscula*) were recorded from both the museum and field surveys. Therefore, a total of 36 siluriform species are currently known from the river. Of these, one we were unable to determine (*Pimelodella* sp.), and four we suggest could represent undescribed species (Tatia aff. musaica, *Ancistrus* sp. "INPA 43862", *Hypancistrus* sp. "INPA 43863" and *Pseudolithoxus* sp. "INPA 43888").

The checklist is far from complete, however, missing entirely families such the callichthyids and aspredinids, although we sampled habitats most likely to yield these groups, such as igarapés, only superficially. Despite this, we feel that rapid publication of even modest datasets and small-scale surveys can make valuable additions to biodiversity science in the Amazon by both presenting the early discovery of new species and the extension of ranges for known species. The study also highlights the paucity of neotropical ichthyological data in GBIF/GenBank, and makes a small contribution.

### Biogeography

The biogeographic composition of the collection is interesting. While many of the species recorded were cosmopolitan inhabitants of the lowland Amazon—e.g. *Auchenipterichthys longimanus*, *Goeldiella eques*, *Hypoptopoma incognitum*, *Lasiancistrus schomburgkii*, *Loricaria cataphracta*, *Pseudoloricaria laeviuscula*, *Rineloricaria lanceolata*, *Calophysus macropterus*, *Pimelodus blochii*, and *Phractocephalus hemioliopterus*—there are suggestions of connections to more specialised faunas. For example: the presence of *Ancistrus dolichopterus* and *Dekeyseria scaphirhyncha* suggests links to the Rio Negro fauna; *Tatia nigra*, on the other hand, is only known from the neighbouring Uatumã and Trombetas rivers, both south draining rivers of the Guiana Shield; two species—Tatia aff. musaica and the undescribed *Pseudolithoxus* sp.—appear to have some connection with the Río Orinoco; while the presence of *Leporacanthicus galaxias* in the Nhamumdá is strange, and may shed some light on its apparently disjunct distribution in the Rio Tocantins,—a north-draining Brazilian Shield river some 2,000 km to the east—the Río Orinoco (Chamon 2007), and apparently also the Rio Aripuanã, an affluent of the Rio Madeira (Seidel and Evers 2005). Thus, the ichthyofauna of the Rio Nhamundá appears to have some interesting affiliations to several faunal areas. This is perhaps exemplified by the best known fish from the river, the discus (*Symphysodon* spp.). Specimens exported from this locality are famous in the aquarium trade for their phenotypic variability (Bleher 2006). Genetic groups of both the Heckel (*S. discus*) and brown (*S. aequifasciatus*) discus species are found in sympatry in the Rio Nhamundá, an area that may in fact comprise a zone of natural hybridisation between the two species (Amado et al. 2011).

In summary, the Rio Nhamundá may therefore be an important sampling location for uncovering biogeographic patterns of fishes of the Lower Amazon and Guiana Shield. Of more urgent importance, however, is the sensitivity of freshwater ecosystems to anthropogenic activities and the requirement for catchment-specific conservation plans (Castello et al. 2013). Here, even rapid biodiversity surveys are of upmost importance in revealing alpha diversity (Alofs et al. 2014), with these surveys being subsequently used to inform conservation priorities (Nogueira et al. 2010). In particular, further surveys are required in the upper section of the Rio Nhamundá above the cataracts, an area that may well harbour endemic or range-restricted species (Alofs et al. 2014).

## Supplementary Material

Supplementary material 1 R script to access GBIF and FishBase records Data type: Plain text file (R script)
File: oo_42806.R Rupert A. Collins

Supplementary material 2 Bash script to search locally stored checklists in PDF format Data type: Plain text file (bash script)
File: oo_42807.sh Rupert A. Collins

XML Treatment for Auchenipteridae

XML Treatment for Ageneiosus sp. "INPA 33873"

XML Treatment for Ageneiosus ucayalensis

XML Treatment for Auchenipterichthys longimanus

XML Treatment for Tatia musaica

XML Treatment for Tatia nigra

XML Treatment for Trachycorystes trachycorystes

XML Treatment for Doradidae

XML Treatment for Astrodoras asterifrons

XML Treatment for Hassar orestis

XML Treatment for Ossancora asterophysa

XML Treatment for Scorpiodoras heckelii

XML Treatment for Heptapteridae

XML Treatment for Goeldiella eques

XML Treatment for Pimelodella sp.

XML Treatment for Loricariidae

XML Treatment for Hypoptopomatinae

XML Treatment for Hypoptopoma incognitum

XML Treatment for Hypostominae

XML Treatment for Ancistrus dolichopterus

XML Treatment for Ancistrus sp. "INPA 43862"

XML Treatment for Dekeyseria scaphirhyncha

XML Treatment for Hypancistrus sp. "INPA 43863"

XML Treatment for Hypostomus carinatus

XML Treatment for Hypostomus macushi

XML Treatment for Hypostomus plecostomus

XML Treatment for Lasiancistrus schomburgkii

XML Treatment for Leporacanthicus galaxias

XML Treatment for Peckoltia vittata

XML Treatment for Pseudolithoxus sp. "INPA 43888"

XML Treatment for Loricariinae

XML Treatment for Farlowella nattereri

XML Treatment for Limatulichthys griseus

XML Treatment for Loricaria cataphracta

XML Treatment for Pseudoloricaria laeviuscula

XML Treatment for Rineloricaria lanceolata

XML Treatment for Pimelodidae

XML Treatment for Calophysus macropterus

XML Treatment for Phractocephalus hemioliopterus

XML Treatment for Pimelodus blochii

XML Treatment for Pinirampus pirinampu

XML Treatment for Pseudoplatystoma reticulatum

XML Treatment for Pseudopimelodidae

XML Treatment for Batrochoglanis villosus

XML Treatment for Trichomycteridae

XML Treatment for Pygidianops amphioxus

## Figures and Tables

**Figure 3a.**
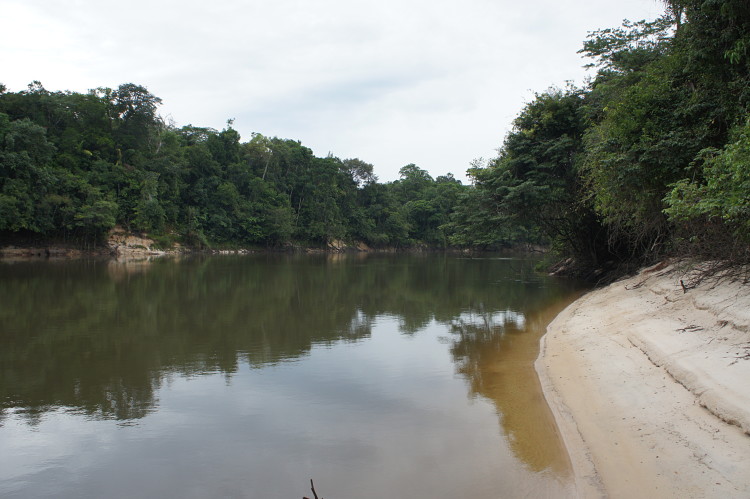
Narrow upstream section near sample site NH05.

**Figure 3b.**
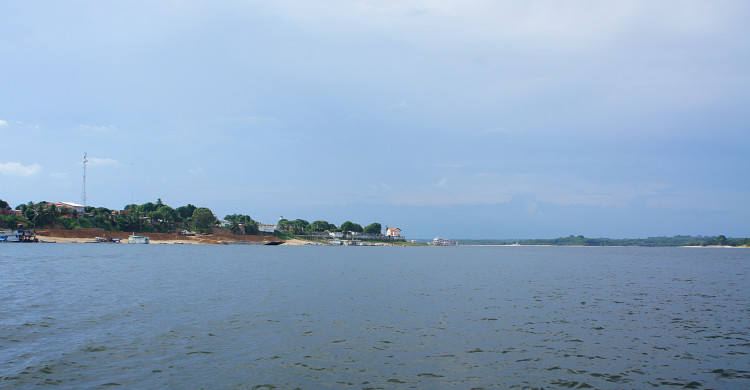
Lake-like lower section at the town of Nhamundá, near sample site NH01.

**Figure 4a.**
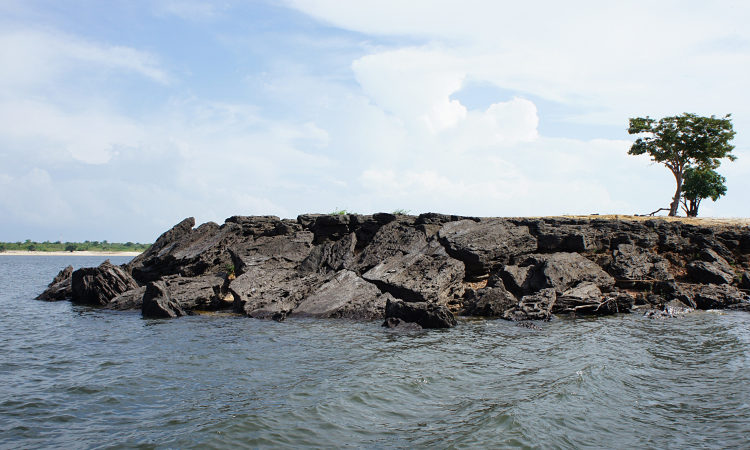
Exposed laterite boulders at sampling site NH01.

**Figure 4b.**
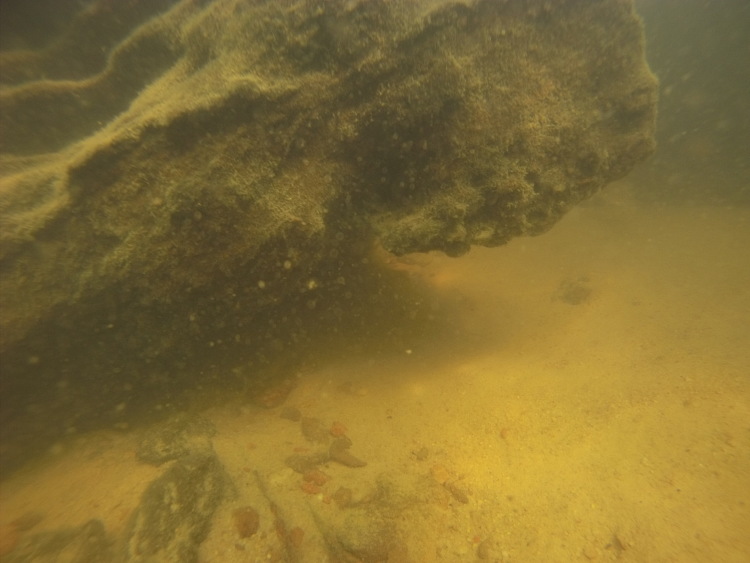
Underwater view of rocks and substrate at sampling site NH01.

**Figure 4c.**
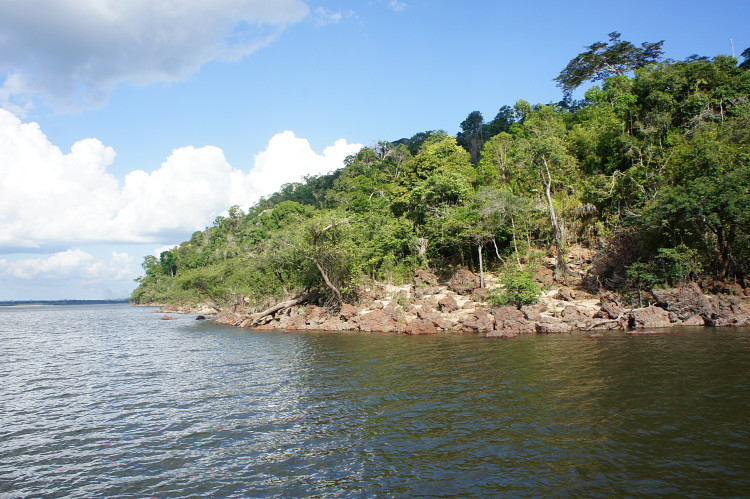
Exposed laterite boulders at sampling site NH12.

**Figure 4d.**
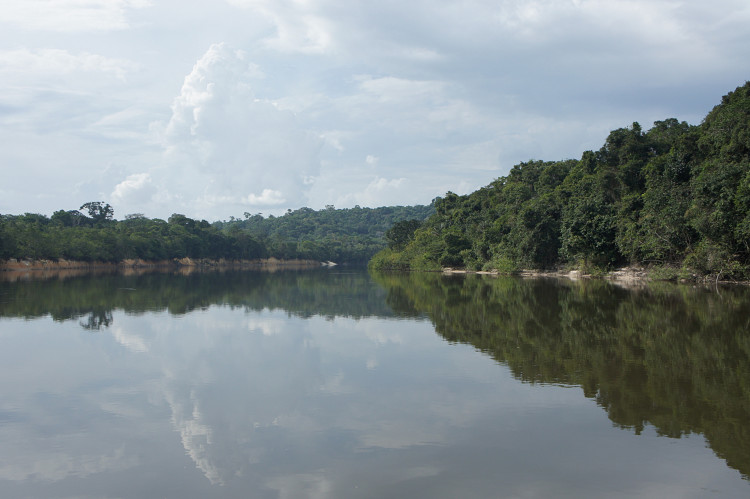
River near sampling site NH04.

**Figure 4e.**
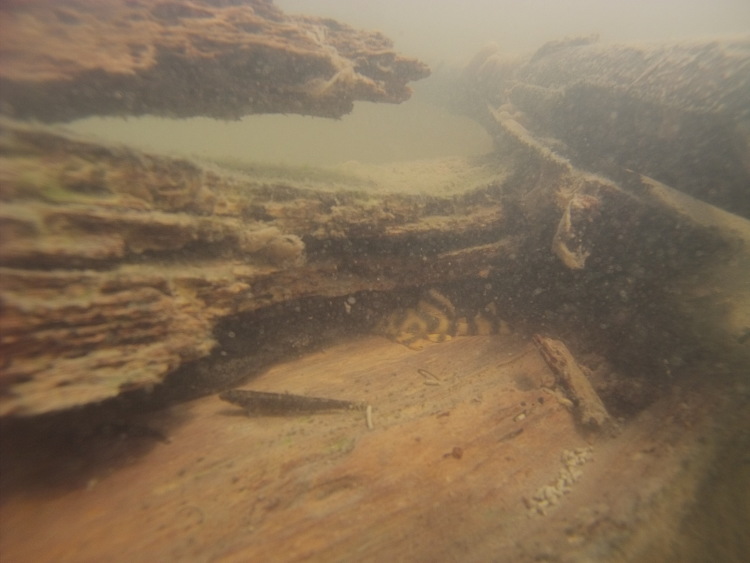
Underwater view of woody substrates at sampling site NH04 (with Peckoltia cf. vittata visible).

**Figure 4f.**
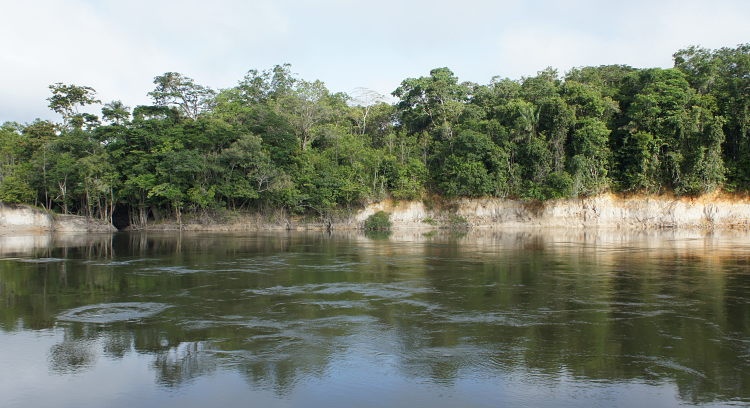
Fast current flowing over exposed bedrock at sampling site NH08.

**Figure 15a.**
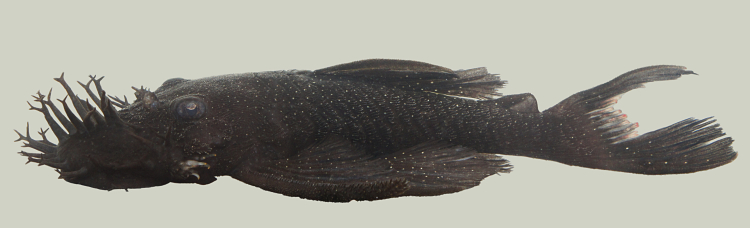
Lateral view.

**Figure 15b.**
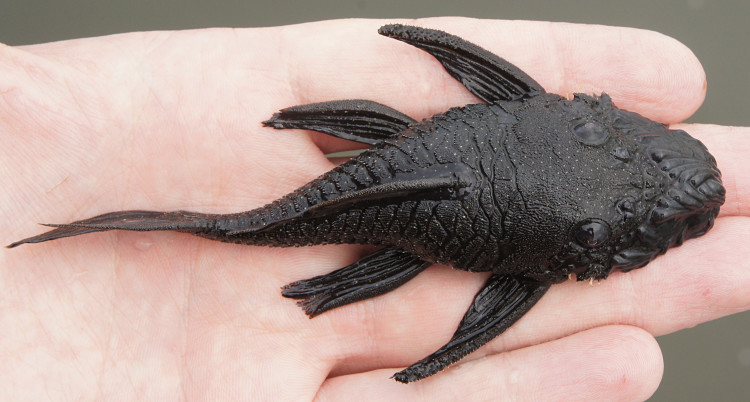
Dorsal view showing body shape.

**Figure 17a.**
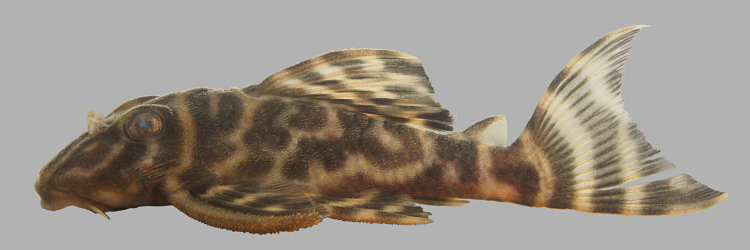
Lateral view, 68.6 mm SL.

**Figure 17b.**
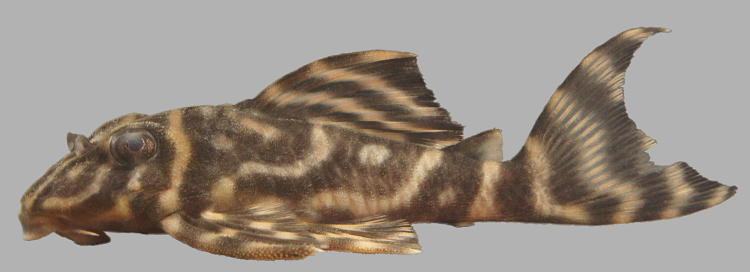
Lateral view, 60.8 mm SL.

**Figure 17c.**
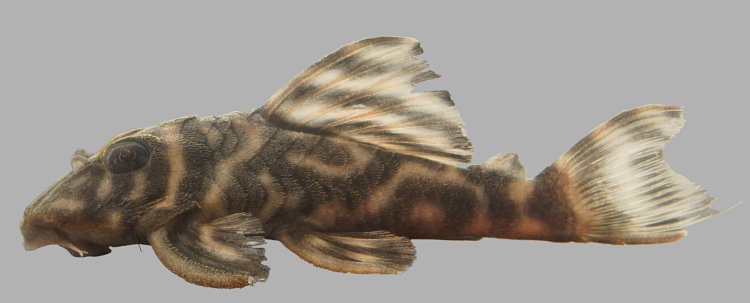
Lateral view, 73.1 mm SL. Image flipped horizontally.

**Figure 17d.**
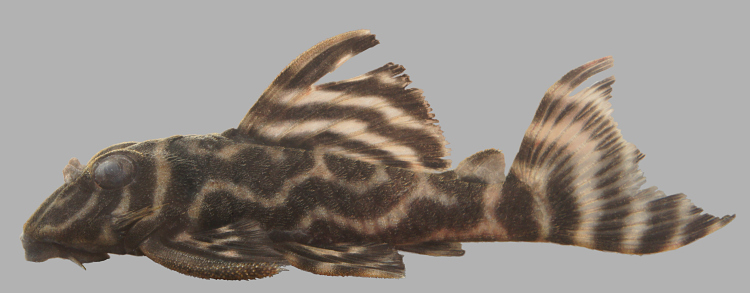
Lateral view, 64.3 mm SL.

**Figure 22a.**
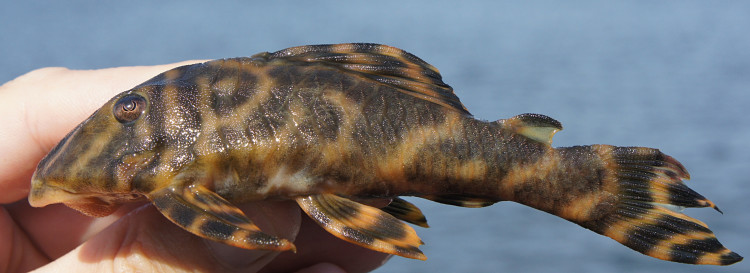
Lateral view (102.5 mm SL; INPA 43869; UFAM:CTGA:14546).

**Figure 22b.**
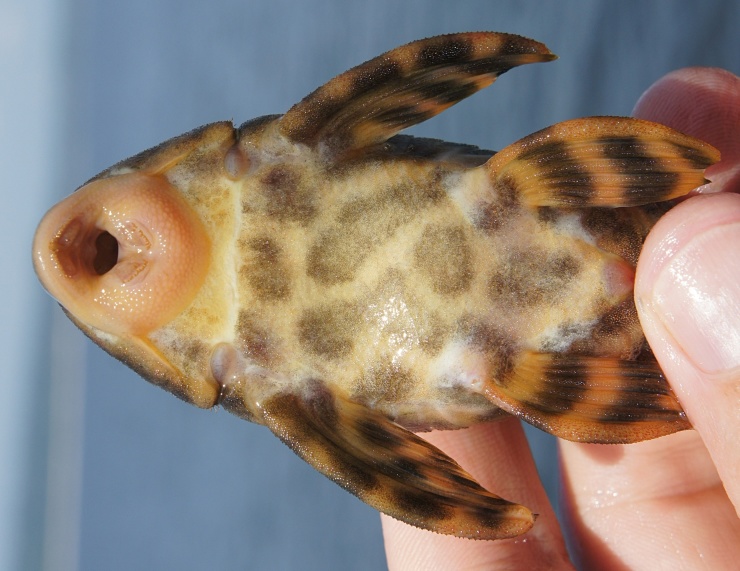
Ventral view (102.5 mm SL; INPA 43869; UFAM:CTGA:14546).

**Figure 22c.**
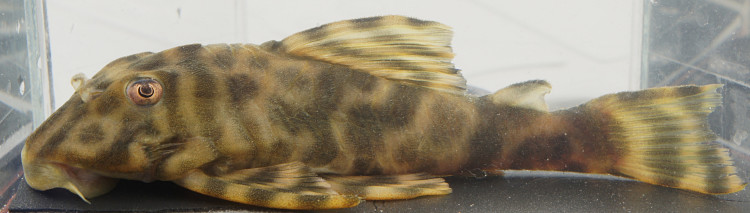
Lateral view (99.7 mm SL; INPA 43894; UFAM:CTGA:14334).

**Figure 22d.**
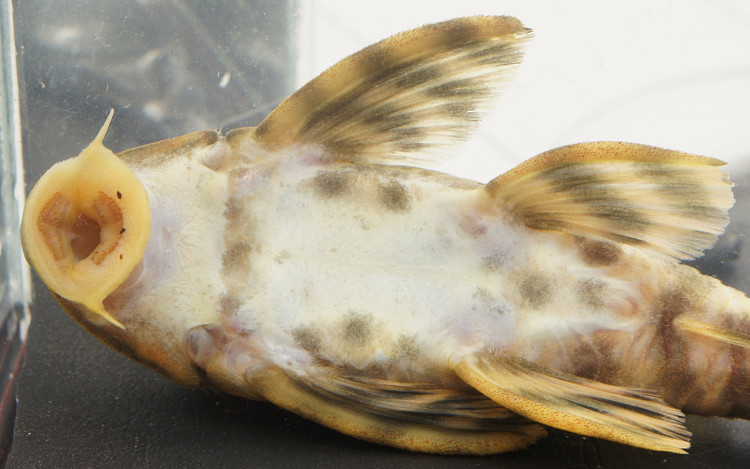
Ventral view (99.7 mm SL; INPA 43894; UFAM:CTGA:14334).

**Figure 22e.**
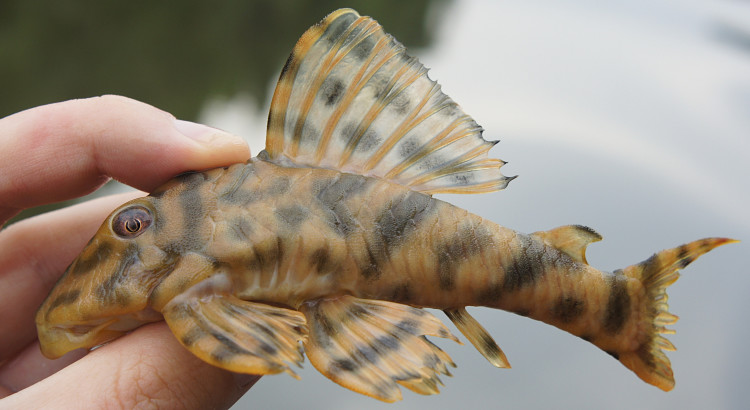
Lateral view (115.6 mm SL; INPA 43881; UFAM:CTGA:14316).

**Figure 22f.**
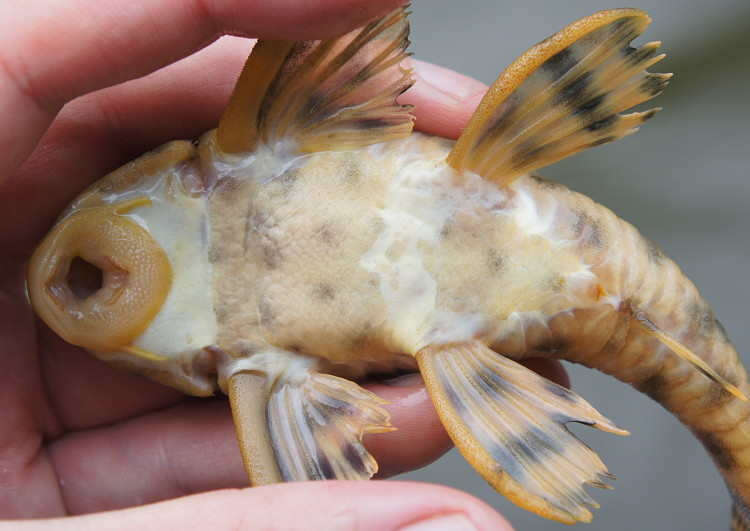
Ventral view (115.6 mm SL; INPA 43881; UFAM:CTGA:14316).
